# Methodological heterogeneity and equity challenges of distributional cost-effectiveness analysis in healthcare: a systematic review from 2017 to 2025

**DOI:** 10.1186/s13690-026-01908-0

**Published:** 2026-04-16

**Authors:** Xinyue Yuan, Jiaqi Shi, Qingqiu Wang, Ming Hu

**Affiliations:** West China School of Pharmacy, Sichuan University, Chengdu, Sichuan 610041 China

**Keywords:** Distributional cost-effectiveness analysis, Health equity, Equity weighting, Healthcare policy

## Abstract

**Background:**

Health equity has become a key focus of global health policy. Distributional cost-effectiveness analysis (DCEA) is one of the most commonly used methods for incorporating health equity into health economics evaluations. However, systematic reviews specifically on DCEA in healthcare remain scarce, and its research methods, findings, and quality have not been clearly summarized.

**Methods:**

A systematic review was conducted in accordance with PRISMA 2020 guidelines, drawing from PubMed (MEDLINE), Web of Science, the Cochrane Library, CNKI, Wanfang, and VIP from inception to 30th August 2025. We included empirical studies in healthcare field that explored the costs and health outcomes of at least two healthcare interventions through DCEA. A total of 923 articles were identified, of which 28 studies met the eligibility criteria. The Quality of Health Economic Studies (QHES) and Consolidated Health Economic Evaluation Reporting Standards (CHEERS) 2022 were both used to assess the quality of the literature. An inductive content analysis was employed to extract detailed research characteristics, identify methodological and outcome differences in DCEA.

**Results:**

All studies were of high quality based on QHES. Relevant research was highly concentrated in developed regions (75.00%), primarily North America and Europe. Infectious diseases (28.57%) and cancers (21.43%) were the most common disease domains. Markov models (25.00%) and Micro-simulation models (21.43%) were the most widely used. Subgroup division mainly relied on societal and structural variables (46.43%) and race and ethnicity (32.14%) as the core basis for equity analysis. The Atkinson index was the most common equity indexes. 85.71% reported win-win interventions, while only one yielded a lose‑lose outcome; a further six studies identified trade‑offs between equity and efficiency.

**Conclusion:**

We elaborated on three key methodological distinctions in DCEA: the basis for subgroup classification, modeling methodology and equity indexes. We identified the following challenges in conducting DCEA: a lack of certified equity subgroup classification criteria and measurement frameworks; difficulties in obtaining evidence for key equity parameters; and policymakers’ unfamiliarity with the method and insufficient emphasis on prioritizing equity considerations.

**Trial registration:**

The protocol and search strategy were registered and published in the PROSPERO International Prospective Register of Systematic Reviews (CRD420251169448).

**Supplementary Information:**

The online version contains supplementary material available at 10.1186/s13690-026-01908-0.

**Table:** 

Text box 1. Contributions to the literature
• Standardized DCEA frameworks are urgently needed to provide actionable pathways for health equity informed decision-making.
• Clarifies key sources of methodological heterogeneity in DCEA, particularly in subgroup classification, modeling methodology and equity index.
• Systematically synthesizes efficiency-equity outcomes from empirical DCEA studies in healthcare when literature accumulation is sufficient.
• Identifies critical implementation barriers, such as the absence of standardized equity subgroup criteria and gaps in equity-relevant evidence, while exploring promising approaches like Aggregate distributional cost-effectiveness analysis (A-DCEA).

## Introduction

The World Health Organization (WHO) defines equity as “the absence of avoidable or remediable disparities between different population groups, whether social, economic, demographic or geographically defined” and strongly advocates for reducing the equity gap in healthcare [1]. The Global Health Strategy 2025–2028 [2] addresses the vulnerabilities of healthcare systems, unequal vaccine distribution, and inadequate global public health governance mechanisms exposed during the Coronavirus Disease 2019 (COVID-19) pandemic, proposing to narrow the gap in accessibility of healthcare services and ensure that all populations have access to affordable and high-quality healthcare services. Currently, there is a growing consensus among regional government agencies to incorporate equity considerations into health technology assessments [3, 4]. Relevant institutions have developed frameworks to guide the consideration of equity issues in health technology assessments, and there are calls for a more formal inclusion of equity impact assessments in health technology assessment decisions [4].

Cost-effectiveness analysis (CEA), one of the most commonly used methods of economic evaluation in healthcare, aims to maximize health gains from limited resources by comparing the costs and health outcomes of alternative interventions [5]. CEA can be implemented using various modeling techniques, such as Markov models [6], Micro-simulation models [7], decision trees [8], and discrete event simulations [9]—to simulate disease progression and estimate costs and health outcomes. Since 2000, international scholars, mainly from the UK and the US, have proposed equity-informative economic evaluations based on traditional CEA [10]. The International Society for Pharmacoeconomics and Outcomes Research (ISPOR) working group points out that incorporating the concept of health equity into CEA is an important emerging direction in the field of health economics [11]. This aims to further consider the equity impact of different equity-influencing factors (such as socioeconomic status or disease severity) on alternative interventions. In recent years, numerous reviews have indicated that equity considerations are increasingly being incorporated into CEA [1]. A review study [12] pointed out that among the applications of the concept of health equity in CEA, DCEA, equity-based weighting method (EWM), Extended cost-effectiveness analysis (ECEA), mathematical programming (MP), and Multi-criteria decision analysis (MCDA) are the most discussed methods. Another review showed that [13], when incorporating health equity considerations into the economic evaluations of health interventions in low-and middle-income countries (LMICs), ECEA, DCEA, subgroup analysis, and Gini coefficient measurement are the main methods used.

**Fig. 1:**
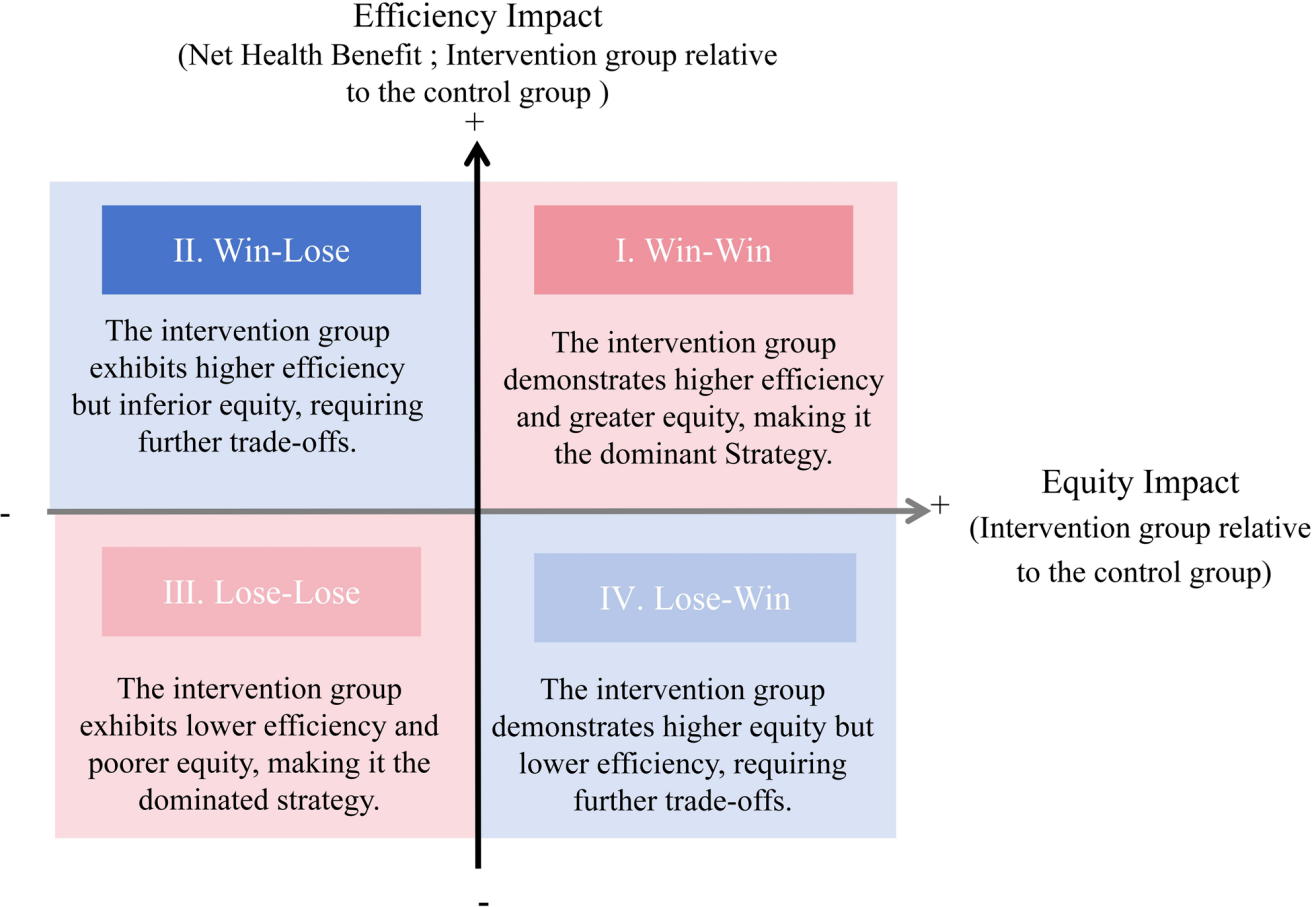
Equity-efficiency impact plane for distributional cost-effectiveness analysis [14–16]. The four quadrants illustrate the trade-off between efficiency (change in population health) and equity (change in health equality). Quadrant I (win-win): the intervention group demonstrates higher efficiency and greater equity, making it the dominant strategy. Quadrant II (win-lose): the intervention group exhibits higher efficiency but inferior equity, requiring further trade-offs. Quadrant III (lose-lose): the intervention group exhibits lower efficiency and poorer equity, making it the dominated strategy. Quadrant IV (lose-win): the intervention group demonstrates higher equity but lower efficiency, requiring further trade-offs.

DCEA was first formally proposed in 2015 and is a framework that incorporates health equity considerations into the economic evaluation of health sector interventions. As an extension of CEA, it can be used to analyze the trade-off between “efficiency” (maximizing the health of the overall population) and “health equity” (achieving a more equitable distribution of health among selected equity strata and the general population), i.e., the “equity-efficiency trade-off,” as shown in Fig. 1 [14–16]. DCEA first constructs a "baseline health distribution", then estimates the distribution of health benefits and costs across different populations, and finally measures changes in health inequality before and after intervention [16, 17]. Changes in health inequality are primarily quantified through social welfare functions (SWFs), which incorporate an inequality aversion parameter [18] to reflect societal preferences for equity. One of the most widely used welfare-based inequality measures is the Atkinson index, which uses the inequality aversion parameter ε to quantify the social psychological dimension of inequality aversion—the larger the value of ε, the more sensitive the index is to disparities affecting lower-income groups [19]. Its subjectivity and variability are relatively strong [20]. Equally Distributed Equivalent Health (EDEH) is calculated based on the Atkinson index, which describes the distributional value of health among the quintiles of IMD [21]. Thus, DCEA can estimate the distribution of health impacts across different social groups (such as groups categorized by socioeconomic status or ethnicity), enabling healthcare decision-makers to assess the equity impact of healthcare decisions quantitatively [18]. Moreover, it can clarify value judgments, and employ sensitivity analysis to evaluate alternatives, making it an ideal methodological approach [12, 22].

A complete DCEA typically requires extensive epidemiological, cost, and utility parameter data at the subgroup level, which is often difficult to fully obtain in real-world scenarios [23]. To address this limitation, researchers have further proposed Aggregate Distributional Cost-Effectiveness Analysis (A-DCEA) in 2018 as a simplified, practical alternative to DCEA [24]. This method starts with the average benefit determined by CEA, expands according to the size of the target population, and then uses social patterns reflected in healthcare use data for the specific disease being addressed to segment the overall benefit for different groups [24].

DCEA has seen its methodological value become increasingly prominent with the accumulation of relevant evidence, making it necessary to conduct a systematic review to integrate and analyze its methodology and outcomes. Existing systematic reviews (including conference abstracts) [10, 25–27] that mentioned DCEA still present several limitations. First, they focus primarily on describing the basic concepts, application contexts, and general analytical procedures of DCEA, with limited synthesis of study outcomes and insufficient consolidation of methodological approaches. Second, the quality assessment of included studies has typically relied on a single or narrow set of tools, without the combined use of reporting tools and critical appraisal tools to separately evaluate reporting completeness and risk of bias. As DCEA evidence has continued to accumulate, there is a clear need for an updated systematic review that addresses these gaps.

Therefore, this study aims to systematically identify and assess empirical research on DCEA in the field of healthcare, with a particular focus on conducting an in-depth analysis and synthesis of three methodological dimensions that have not been centrally addressed in previous research: the rationale for subgroup classification, modeling methodology, and the selection of equity measurement indexes. Furthermore, by synthesizing efficiency-equity trade-off results, we examine the heterogeneity in methodologies and findings, the current challenges faced, and potential strategies to address them.

## Methods

This systematic review follows the Preferred Reporting Items for Systematic reviews and Meta-Analysis (PRISMA) guidelines [28]. We used the PRISMA 2020 checklist to ensure we covered all entries in an evidence-based manner (Supplemental Table 1). The protocol has been agreed and registered in PROSPERO (*protocol number CRD420251169448*) prior to the start of this specific review on DCEA.

### Search strategy

To ensure comprehensive coverage, a systematic search strategy was employed, utilizing databases such as PubMed (MEDLINE), Web of Science, and the Cochrane Library as primary databases for literature retrieval, supplemented by Chinese databases including CNKI, Wanfang, and VIP. The search was conducted on 30th August 2025, without time and language restrictions. The search strategy combined subject terms and free terms. Search terms included: “distributional cost effectiveness analysis”, “DCEA”, “distributional economic evaluation”, “equity”, “inequality”, “fairness”, and “opportunity cost”. These search terms were selected through a thorough iterative process involving repeated additions, modifications, and deletions of search terms and term combinations, based on preliminary broad reviews and reference to other literature [10, 12, 27]. To ensure comprehensiveness, a Boolean logic operator (AND/OR) combination search strategy was adopted. Specific search strategies for each database were detailed in Supplemental Table 2.

### Inclusion and exclusion criteria

Inclusion criteria: Empirical literature on DCEA in the fields of healthcare, — that was, original research studies applying DCEA to analyze the costs and health outcomes of two or more alternative medical interventions using observed or modeled quantitative data, rather than being limited to qualitative analysis of definitions and methods, regardless of target population, control group, or the direction/magnitude of the reported results.

Exclusion criteria: (1) Literature not in the fields of healthcare, (2) non-empirical articles that were limited to qualitative analysis of definitions and methods, unable to accurately obtain quantitative data or lacking quantitative data, (3) Analyses that only presented results stratified by relevant equity strata (akin to subgroup analysis), (4) secondary studies such as systematic or narrative reviews, (5) conference summaries, commentary articles, (6) those for which full text could not be obtained.

Records retrieved from database searches were imported into Endnote. A team of researchers (XY, JS, and QW) independently screened records in duplicate based on title and abstract to identify potentially relevant studies. Conflicts were resolved through discussion (XY, JS). Subsequently, full-text articles of potentially relevant studies were retrieved and independently assessed for eligibility by two reviewers (XY, JS) against inclusion criteria. Reasons for exclusion were noted. Disagreements between individual reviewers’ judgments were resolved through discussion and consensus achieved with a third reviewer (MH).

### Quality assessment

The quality appraisal stage was conducted to ensure that the selected studies adhered to rigorous methodological standards and provided reliable findings. As no critical appraisal tool specifically designed for DCEAs currently exists, this study employed two established health economics evaluation frameworks: Consolidated Health Economic Evaluation Reporting Standards (CHEERS) 2022 [29] and Quality of Health Economic Studies (QHES) [30]. CHEERS establishes a detailed set of standards to assess the quality of reports themselves, aiming to enhance clarity and transparency for readers. QHES, meanwhile, possesses strong discriminant validity, assisting researchers in identifying and selecting high-quality literature.

The CHEERS 2022 scale consists of 7 parts: (1) Title, (2) Abstract, (3) Introduction, (4) Methods, (5) Results, (6) Discussion, and (7) Other Relevant Information, containing 28 items with accompanying descriptions **(**Supplemental Table 3**)**. The quality of the included studies was assessed according to the criteria listed in the CHEERS checklist. “Yes” was used to indicate compliance with the criteria, and “No” was used to indicate non-compliance. The number of “Yes” entries was converted into a percentage to evaluate the quality of the studies [29].

The QHES is a validated and reliable quantitative measurement tool designed to evaluate the methodology, validity, and transparency of health economic evaluations [30, 31]. The tool is composed of 16 binary questions (i.e., answered yes or no) that evaluate several important elements required for a high-quality health economic evaluation (Supplemental Table 5). Each question has an assigned score value ranging from 1 to 9, where questions answered “yes” receive the full point value. Questions answered “no” receive no points. The question scores are summed to obtain a final summary score ranging from 0 to 100 points, where higher scores represent higher quality. Studies were also classified into 1 of 4 quartiles based on the QHES total score: high quality (75–100); fair (50–74); poor (25–49); or extremely poor (0–24) [32].

Each study underwent independent evaluation based on the QHES and CHEERS 2022 checklists. To ensure consistency in reviewers’ interpretation of issues, we conducted a pilot test on 5 studies prior to completing all QHES and CHEERS assessments. Three reviewers (XY, JS, and QW) conducted the assessments independently, with any discrepancies resolved through discussion and consensus with a fourth reviewer (MH).

### Data extraction strategy

After assessing the quality of the included studies, data extraction was carried out with a standardized data extraction checklist. The checklist underwent an iterative refinement process involving three rounds of discussion within the author group until a consensus was reached on the final version. The checklist includes: (1) general information (author, publication year, country, research perspective); (2) study population, disease domains, intervention and control group; (3) study methodology (subgroup classification criteria, equity index, modeling techniques, sensitivity analysis); and (4) health economic outcomes (base-case CEA results, health outcome measurement indicators, equity analysis results).

Records retrieved from database searches were imported into Microsoft Excel, where an extraction matrix was created. Data extraction was carried out independently by three reviewers (XY, JS, and QW). Any disagreements between reviewers during the screening process were resolved through discussion until consensus was reached, with a fourth reviewer consulted when necessary.

### Data synthesis

The DCEA measures the overall health impact as “incremental Net health benefit (iNHB)” and the health inequality impact as “difference between population increment Equally distributed equivalent health (iEDEH) and population iNHB”. When iEDEH > iNHB, the equity impact is positive [16]. The DCEA uses an equity-efficiency diagram (**see** Fig. 1) similar to traditional cost-effectiveness analysis to weigh these two objectives. The options in the first and third quadrants are easier to identify, which do not require sacrificing the value of one dimension to obtain the value of another. The options in the second and fourth quadrants indicate that it is impossible to achieve both goals of improving equity and average health. It is necessary to use a method to quantitatively measure the value of equity and combine it with the efficiency dimension to assess the relative merits of the overall well-being level.

To assess the direction of equity-related evidence, we classified the included studies based on their conclusions regarding the CEA results and preferences for equity. First, following the approach employed by a recent review [10, 33], we assessed the basecase cost-effectiveness of each intervention. Studies were categorized as: (1) “cost-effective” if the incremental cost-effectiveness ratio (ICER) of at least one evaluated intervention was at or below a stated threshold; (2) “dominant” if the intervention had negative incremental costs and positive incremental benefits; (3) and “not cost-effective” if all evaluated interventions had ICERs above a stated threshold. Second, we further classified studies based on their location on the equity-efficiency impact plane (see Fig. 1):(1) Win-win (first quadrant); (2) lose-lose (third quadrant), and (3) trade-offs between equity and efficiency (second and fourth quadrants: gains in one dimension come at the expense of another). For studies not explicitly categorizing these outcomes, classification was inferred based on the direction of net health benefit changes and the significance of inequality impacts.

## Results

### Search results

**Fig. 2:**
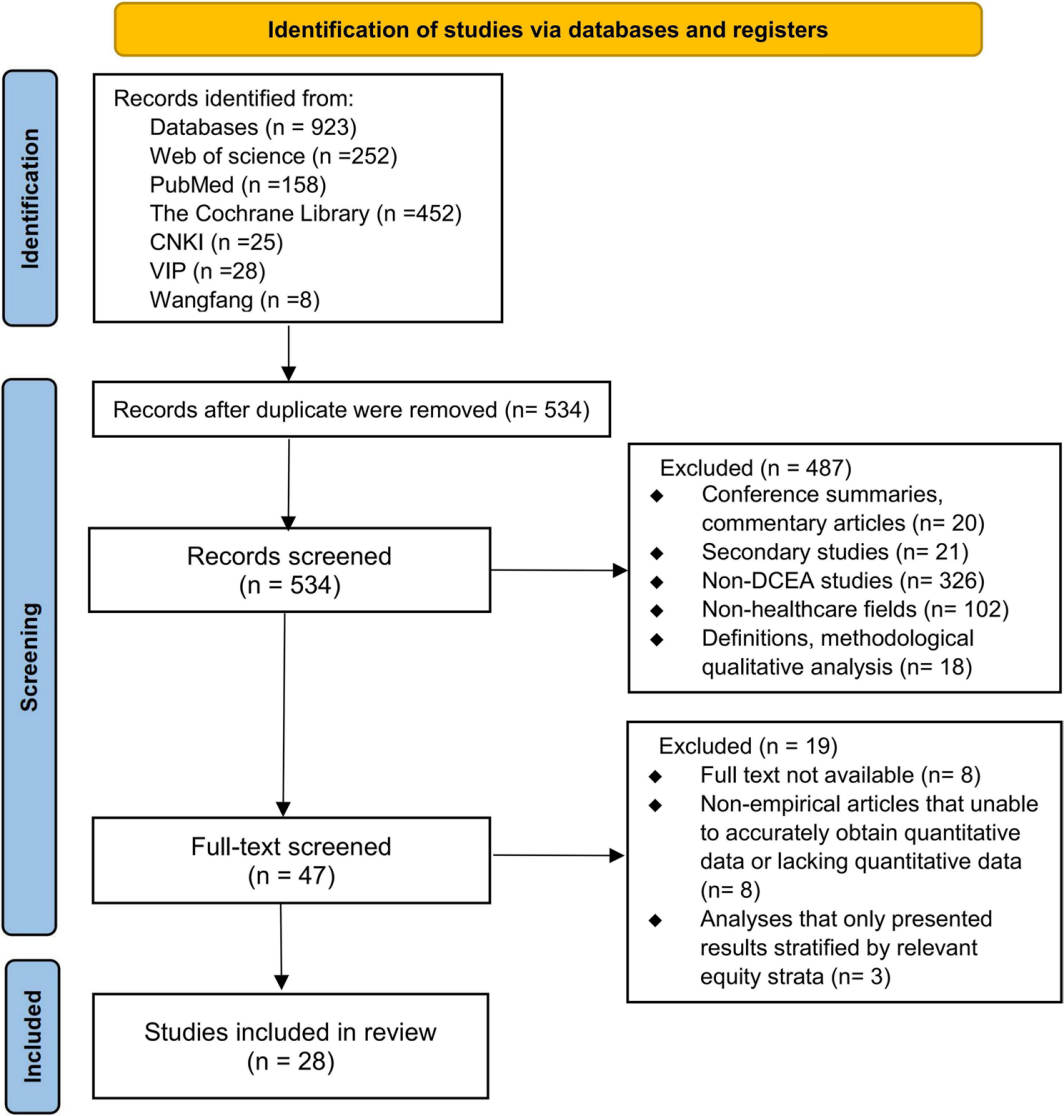
Study selection for systematic review of empirical studies that applied distributional cost-effectiveness analysis to evaluate the costs and health outcomes of at least two healthcare interventions published from inception to 30th August 2025 using PubMed (MEDLINE), Web of Science, the Cochrane Library, CNKI, Wanfang, and VIP databases. Diagram based on the PRISMA 2020 flow diagram. PRISMA: Preferred Reporting Items for Systematic Reviews and Meta-Analyses

Following the search strategy, 923 citations were identified in total. After removing duplicates, 534 unique records were retained. These records were screened for relevance, leading to the exclusion of 487 documents. Subsequently, the remaining 47 articles underwent critically appraised for eligibility, and 19 were excluded for the following reasons: Analyses that only presented results stratified by relevant equity strata (*n* = 3), non-empirical articles that were unable to accurately obtain quantitative data or lacking quantitative data (*n* = 8), and full text was not available (*n* = 8). Finally, a total of 28 studies were included, all of which were in English. The literature search process and results were shown in Fig. 2.

### Study characteristics

**Fig. 3:**
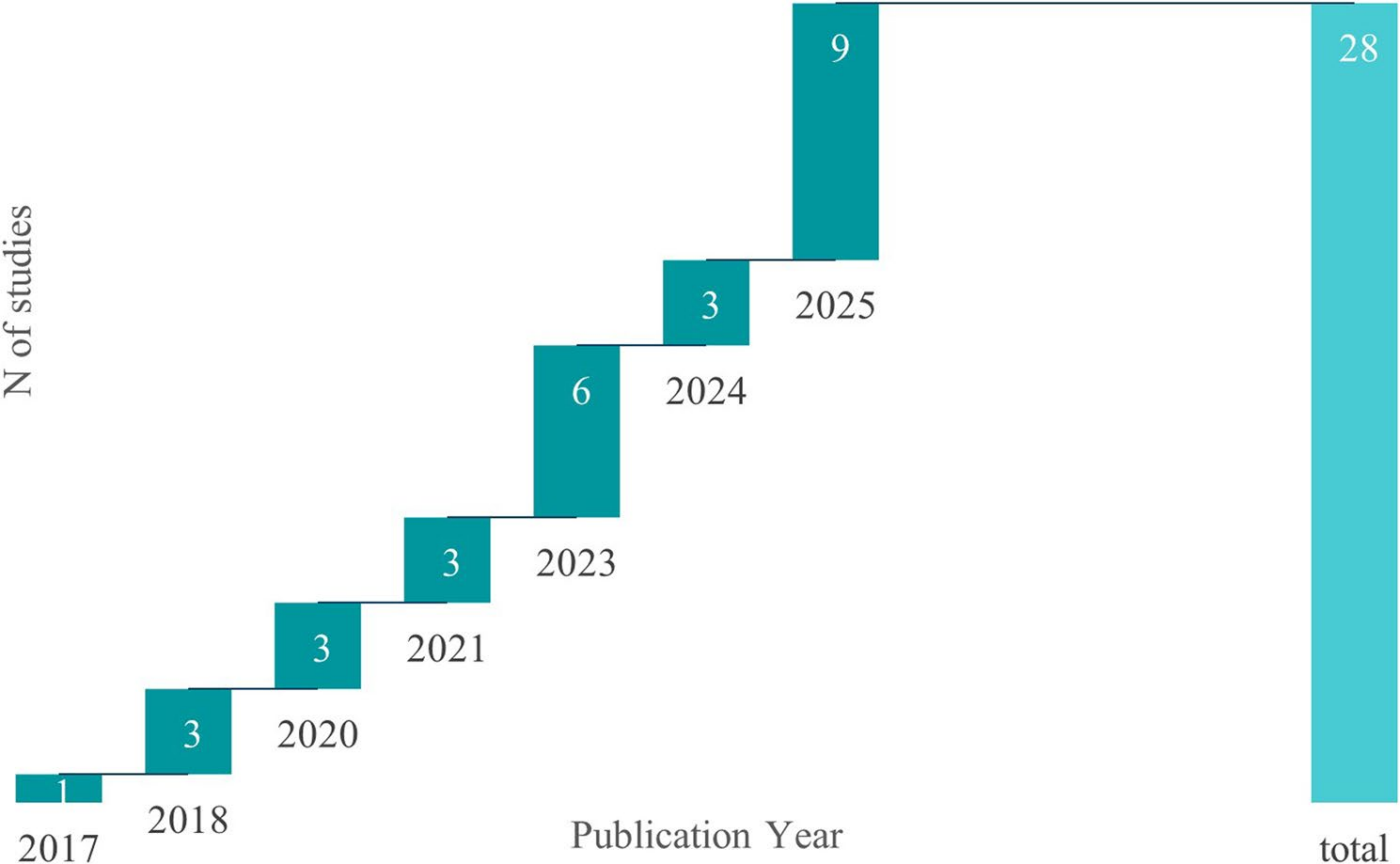
Annual publication trends of the 28 included empirical studies that applied distributional cost-effectiveness analysis to evaluate the costs and health outcomes of at least two healthcare interventions, published from 2017 to 2025

The number of empirical studies on DCEA in the healthcare field varies from year to year (Fig. 3). Prior to 2020, there were a total of four (14.28%) studies [34–37], while twenty-four studies [38–61] were conducted after 2020 (after the COVID-19 pandemic), accounting for as high as 85.71%, with year-to-year fluctuations (three in 2020, three in 2021, six in 2023, three in 2024) and reaching a marked peak of nine studies in 2025.

**Fig. 4:**
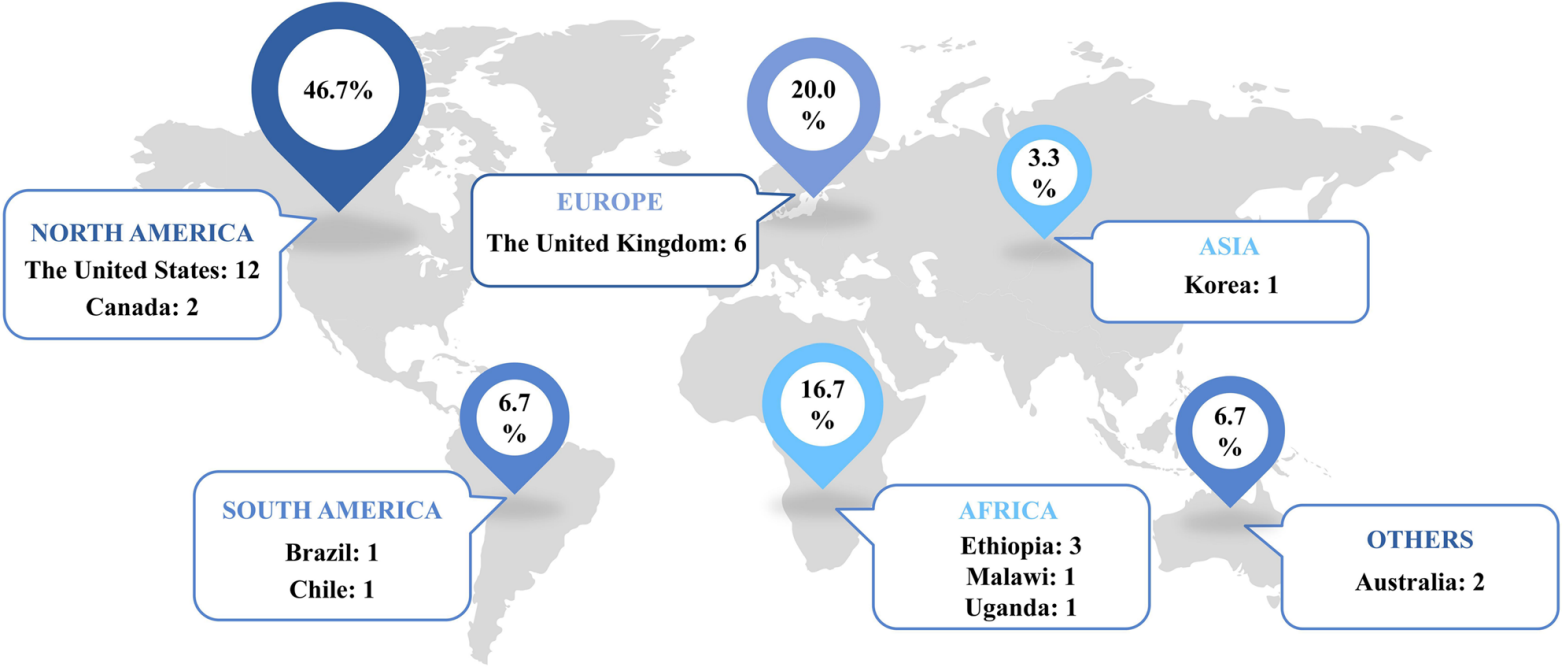
Geographic distribution of the 28 included empirical studies that applied distributional cost-effectiveness analysis to evaluate the costs and health outcomes of at least two healthcare interventions, published from 2017 to 2025

As shown in Fig. 4, 21 articles were primarily applied in developed regions such as North America (mainly the United States) [37, 42, 44, 46, 51–53, 55, 57–60], Europe (the United Kingdom) [39, 40, 45, 49, 50, 54], Australia [48, 61] and Asia (South Korea) [36]. Seven studies were conducted in developing countries, such as Ethiopia [35, 43, 47], Uganda [34], Malawi [38] in Africa, and Brazil [41] and Chile [56] in South America. More than half (60.71%) of the studies used the patients [37, 45, 46, 50, 51, 53, 56, 59, 60] or general population [37, 38, 44, 47, 52, 54, 55] as the research population, followed by adults [39, 40, 42, 56, 61] and children [35, 43, 48, 58]. 3 studies [38, 40, 41] focused on multiple diseases, with 8 articles [35, 42–44, 47, 53, 54, 61] evaluating interventions for infectious diseases, including COVID-19 [44, 53, 61], Human immunodeficiency virus (HIV) [42, 47], virus-induced diarrhea [35, 47], and vaccine-preventable diseases (specifically Serogroup B invasive meningococcal disease) [54]. 6 articles focused on cancer, including non-metastatic castration-resistant prostate cancer [59], cervical cancer [34, 36], and lung cancer [40, 45, 60]. 2 articles [54, 58] adopted multiple research perspectives. Half (53.57%) [37–43, 45–47, 51, 56, 59, 61] of the literature adopted a national/local healthcare system perspective, while 6 articles [34, 36, 49, 50, 52, 57] and 6 articles [44, 48, 53–55, 60] focused solely on a societal perspective and a healthcare payer perspective, respectively. No studies adopted a patient or healthcare institution perspective (see Table 1). Of the included studies, 4 articles [38, 45, 50, 56] used A-DCEA. For detailed information on multiple perspectives and multiple diseases, as well as other key characteristics of the reviewed DCEA studies, please refer to Supplemental Table 7.

**Table 1:** Basic characteristics of the 28 included empirical studies that applied distributional cost-effectiveness analysis to evaluate the costs and health outcomes of at least two healthcare interventions, published from 2017 to 2025

Item	Number	Percent (%) ^a^	References
Study Regions			
North America	12	42.86%	[37, 42, 44, 46, 51–53, 55, 57–60]
South America	2	7.14%	[41, 56]
Europe	6	21.43%	[39, 40, 45, 49, 50, 54]
Africa	5	17.86%	[34, 35, 38, 43, 47]
Asia	1	3.57%	[36]
Australia	2	7.14%	[48, 61]
Study Population			
General population	8	28.57%	[37, 38, 41, 44, 47, 52, 54, 55]
Children	4	14.29%	[35, 43, 48, 58]
Adults	5	17.86%	[39, 40, 42, 56, 61]
Females	2	7.14%	[34, 36]
Elderly	1	3.57%	[49]
Patients	9	32.14%	[37, 45, 46, 50, 51, 53, 56, 59, 60]
Disease Domain			
Cancer	6	21.43%	[34, 36, 37, 45, 59, 60]
Infectious diseases	8	28.57%	[35, 42–44, 47, 53, 54, 61]
Cardiovascular diseases	2	7.14%	[39, 57]
Multiple diseases	3	10.71%	[38, 40, 41]
Others	9	32.14%	[46, 48–52, 55, 56, 58]
Research Perspective			
Whole society	6	21.43%	[34, 36, 49, 50, 52, 57]
Healthcare system	14	50.00%	[37–43, 45–47, 51, 56, 59, 61]
Payers	6	21.43%	[44, 48, 53–55, 60]
Multiple perspectives ^b^	4	14.29%	[39, 40, 54, 58]

Percentages may sum to more than 100% for items where multiple categories apply to a single study. References are numbered as in the main text

^**a**^ Percentages are calculated based on the total number of studies (*N* = 28)

^b^ Collins, et al. (2020) [39], Love-Koh, et al. (2020)[40] that simultaneously adopt both national and local healthcare system perspectives also fall under the category of Multiple perspectives

### Quality assessment results

#### CHEERS 2022

Of the 28 included articles, all literature had a proportion of “yes” items exceeding 78%, with the highest reaching 89%. Among the 28 evaluation items, 18 had a 100% reporting rate (namely: title, abstract, background and objectives, study population, setting and location, perspective, selection of outcomes, measurement of outcomes, valuation of outcomes, measurement and valuation of resources and costs, analysis and assumptions, heterogeneity, characterizing distributional effects, study parameters, summary of main results, limitations, generalizability, source of funding, and conflicts of interest). Two items had a 0% reporting rate, namely health economic analysis plan and the effect of engagement with patients and others affected by the study. Six articles [35, 36, 38, 44, 45, 51] did not specify the time horizon, 10 articles [36–38, 41, 44, 45, 47, 50, 51, 56] had incomplete discount rate information, four articles [44, 45, 50, 51] did not describe currency, price dates and conversion details. Six articles [35, 38, 44, 45, 47, 50] did not clarify the rationale and description of model. Only one article [51] described the approach to engagement with patients and others affected by the study (see Supplemental Table 4 for details).

#### QHES instrument

The mean QHES score of the identified studies was 93.96 ± 6.85 (range, 76–100), which is considered high quality according to the QHES quartiles [32]. All studies (100%) were considered high quality (QHES 75–100). Individual QHES scores for each study were provided in Supplemental Table 6.

### Methodological characteristics

A summary of the methodological characteristics of the included literature was shown in Table 2. Detailed information for methodological characteristics of included articles was listed in Supplemental Table 8.

**Table 2:** Methodological characteristics of the 28 included empirical studies that applied distributional cost-effectiveness analysis to evaluate the costs and health outcomes of at least two healthcare interventions, published from 2017 to 2025

Item	Number	Percent (%) ^a^	References
Subgroup Classification			
IMD	5	17.86%	[39, 40, 45, 50, 54]
SVI	4	14.29%	[44, 52, 53, 55]
SEP/SES	3	10.71%	[48, 49, 56]
Race and ethnicity	11	39.29%	[37, 42, 44, 51–53, 55, 58–61]
HWQ	4	14.29%	[35, 38, 47, 57]
IWI	1	3.57%	[38]
Geographic region	3	10.71%	[36, 41, 43]
Accessibility and screening frequency	1	3.57%	[34]
Disease severity	1	3.57%	[46]
Educational attainment	2	7.14%	[51, 57]
Modeling Analysis			
Markov models	7	25.00%	[36, 40, 41, 43, 46, 52, 61]
Micro-simulation models	6	21.43%	[34, 39, 48, 54, 57, 58]
Decision tree model	1	3.57%	[51]
Partitioned survival model	1	3.57%	[37]
Decision tree-partitioned survival model	1	3.57%	[60]
Decision tree-Markov models	3	10.71%	[53, 55, 56]
Discrete individual simulation model	1	3.57%	[49]
Others	2	7.14%	[42, 59]
Not mentioned	6	21.43%	[35, 38, 44, 45, 47, 50]
Equity Index			
Atkinson index	20	71.43%	[35–38, 40, 41, 44–47, 49–53, 55–57, 59, 60]
Kolm index	5	17.86%	[41, 47, 51, 54, 60]
Gini index	2	7.14%	[43, 51]
Slope inequality index	5	17.86%	[39–41, 47, 48]
Relative inequality index	3	10.71%	[40, 41, 47]
Theil index	1	3.57%	[42]
Difference index	1	3.57%	[42]
Not mentioned	3	10.71%	[34, 58, 61]
Uncertainty Analysis			
Inequality aversion parameters	15	53.57%	[35, 37, 38, 40, 44, 45, 47, 50, 53–57, 59, 60]
Opportunity cost distributions	5	17.86%	[35, 44, 47, 51, 52]
Disease incidence/prevalence	4	14.29%	[38, 46, 47, 56]
Others	8	21.43%	[34, 39, 41, 42, 48, 49, 58, 61]
Not mentioned	2	7.14%	[36, 43]

Percentages may sum to more than 100% for items where multiple categories apply to a single study. References are numbered as in the main text

*IMD* Index of Multiple Deprivation, *SVI* Social Vulnerability Index, *SEP/SES* Socio-Economic Position/Status, *HWQ* Household Wealth Quintile, *IWI* International Wealth Index

^**a**^ Percentages are calculated based on the total number of studies (*N* = 28)

#### Subgroup classification

Antecedents of health equity refer to events that must be in place before the concept can occur. Conditions related to health equity can be categorized into six main antecedent categories: (a) Environmental, (b) Financial and economic, (c) Law, political, and policy, (d) Societal and structural, (e) Research, (f) Digital divide and technology [62]. In the DCEA, it is necessary to determine the baseline health level of the population under no-intervention conditions, analyze the baseline health level and equity factors affecting health benefits, and classify equity subgroups accordingly [63]. This study classified the DCEA subgroups into six categories based on the antecedents of health equity.

Eleven articles [41, 42, 44, 46, 51–53, 55, 57, 58, 61] employed multiple classification methods. Twelve studies included in the study used comprehensive (d) Social and structural variables, such as the England Index of Multiple Deprivation (IMD) [39, 40, 45, 50, 54], Social Vulnerability Index (SVI) [44, 52, 53, 55], and Socioeconomic position /status (SEP/SES) [48, 49, 56]. Another 11 studies used single social variables such as race and ethnicity [37, 42, 44, 51–53, 55, 58–61], gender [58] and age [51, 61] for subgroup analysis, reflecting health differences under identity discrimination/culture and the interaction of social identity and position. Among these, subgroup classification based on race and ethnicity is easily influenced by (e) Research, hence participants from minority and marginalized groups were always excluded in previous studies [64]. Five studies used (b) Financial and economic metrics such as the Household Wealth Quintile (HWQ) [35, 38, 47, 57] and the International Wealth Index (IWI) [38]. Their classification logic is based on the core assumption that “resource availability determines health opportunities,” arguing that economic resources directly influence access to health services, lifestyle, and disease burden, directly quantifying the contribution of economic inequality to health. Three articles [36, 41, 43] used the (a) Environmental dimension of geographic region as the basis for subgroup classification. Additionally, one article each categorized screening accessibility and screening frequency [34], and disease severity [46]. Their core logic was the heterogeneity of intervention accessibility or clinical status, focusing on the equity of intervention implementation processes, such as whether screening covered marginalized groups, or the modifying effect of disease severity on treatment effectiveness, effectively supplementing (d) social factors [10].

#### Modeling analysis

Regarding the type of model used, 6 studies [35, 38, 44, 45, 47, 50] did not mention any model type, with Markov models being the most frequently used (25.00%) [36, 40, 41, 43, 46, 52, 61]. Micro-simulation models (MMs) were the second most frequently used, with 6 studies [34, 39, 48, 54, 57, 58] using it. MMs specifically covering socioeconomic status-specific micro-simulation models (SEP-MMs) and Monte Carlo Markov-chain micro-simulation models (MCMC-MMs). The former can quantify the cost-effectiveness difference of an intervention between the high-income group and the low-income group [48]. The latter, through iterative sampling of MCMC, can calculate the posterior distribution of cost-effectiveness indicators for each subgroup and perform statistical tests on inter-group differences [35, 57].

One study [51] used the Decision tree models. Four studies combined the decision tree model with other models, including one involving Decision tree-partitioned survival models [60] and three involving Decision tree-Markov models [53, 55, 56]. Additionally, one study each used the Discrete Individual Simulation Model (DSM) [49], the Dynamic Room HIV Transmission Model [42], and the Partition Survival Model (PSM) [37].

#### Equity index

In existing empirical studies of DCEA analyzing healthcare projects, the equity of calculating the health levels of each subgroup in the intervention and control groups is mainly achieved through a series of equity measurement functions [16]. Commonly used methods for measuring inequality can be roughly divided into measuring relative inequality (Atkinson index and Relative inequality index), absolute inequality (Kolm index and Slope inequality index), objective equity functions (Gini coefficient, Theil index), and methods for measuring the gap with reference values (Absolute gap index and Relative gap index) [12].

20 studies [35–38, 40, 41, 44–47, 49–53, 55–57, 59, 60] used the Atkinson index to measure inequality. In addition, the Kolm index [41, 47, 51, 54, 60], Gini index [43, 51], Slope inequality index [39–41, 47, 48], Relative inequality index [40, 41, 47], Theil index [42], and Difference index [42] were also used in the included studies. Six studies [40–42, 47, 51, 60] used more than one equity indexes; three studies [34, 58, 61] did not use any equity indexes, indirectly assessing the equity per capita Quality-adjusted life year (QALY) gain gap through differences in health and economic benefits between subgroups.

#### Uncertainty analysis

In DCEA, uncertainty analysis can simultaneously focus on two types of decision uncertainty: the probability that an intervention will improve overall health and the likelihood that it will reduce health inequality [65]. Two studies [36, 43] did not explicitly mention uncertainty analysis, whereas the remaining studies included it. Fifteen studies [35, 37, 38, 40, 44, 45, 47, 50, 53–57, 59, 60] and five studies [35, 44, 47, 51, 52] conducted deterministic sensitivity analyses on inequality aversion parameters and opportunity cost distributions, respectively, parameters crucial for EDEH calculations. Four studies [38, 46, 47, 56] conducted uncertainty analyses on disease incidence/ prevalence.

### Impact of equity adjustments

The base-case cost-effectiveness findings and results of the equity analyses for each study were detailed in Supplemental Tables 8 and summarized in Table 3. Most studies (85.71%) [34–38, 40–45, 47–50, 52–57, 59–61] found that the intervention(s) evaluated in the included studies was (were) cost-effective using commonly used cost-effectiveness thresholds. Dominant interventions were identified in six studies [39, 40, 42, 50, 58, 61] (21.43%), while four studies [35, 45, 46, 48] (14.29%) concluded that the intervention(s) was (were) not cost-effective. After incorporating equity considerations, 85.71% of studies [34, 36–42, 44–50, 52–60] reported that intervention(s) was (were) win-win, whereas only one study [48] indicated a lose-lose outcome. Additionally, six studies [34, 35, 43, 45, 60, 61] examined trade-offs between equity and efficiency: among these, three [34, 60, 61] reported interventions that were cost-effective but harmed equity, and two [35, 37] found interventions that improved equity but were cost-ineffective, and one [43] found both due to different interventions. Notably, one study [61] identified a dominant intervention that was not win-win—it was more efficient but less equitable. Of the studies that deemed interventions cost-effective, 87.50% [34, 36–38, 40–42, 44, 45, 47–50, 52–57, 59, 60] also fell within the win-win category.

**Table 3:** Summary of base-case cost-effectiveness analysis results and equity analysis outcomes of the 28 included empirical studies that applied distributional cost-effectiveness analysis to evaluate the costs and health outcomes of at least two healthcare interventions, published from 2017 to 2025

Item	Number	Percent (%) ^a^	References
Basic-case CEA results			
Intervention/s is/are dominant	6	21.43%	[39, 40, 42, 50, 58, 61]
Intervention/s is/are not cost-effective	4	14.29%	[35, 45, 46, 48]
Intervention/s is/are cost-effective	24	85.71%	[34–38, 40–45, 47–50, 52–57, 59–61]
No change or not applicable	1	3.57%	[51]
Equity analysis results			
Win-win (cost-effective, improve equity)	24	85.71%	[34, 36–42, 44–50, 52–60]
Lose-lose (cost-ineffective, harm equity)	1	3.57%	[48]
Trade-offs between equity and efficiency	6	21.43%	[34, 35, 43, 45, 60, 61]
cost-effective, harm equity	4	14.29%	[34, 43, 60, 61]
cost-ineffective, improve equity	3	10.71%	[35, 43, 45]
No change or not applicable	1	3.57%	[51]

Some studies included multiple interventions or scenario analyses and therefore may present two or more different results for base-case cost-effectiveness and/or equity analysis

Win-win: interventions that are both cost-effective and improve equity. Lose-lose: interventions that are not cost-effective and harm equity. Trade-offs: interventions that improve one dimension at the expense of the other

^**a**^ Percentages are calculated based on the total number of studies (*N* = 28)

## Discussion

In recent years, systematic reviews on DCEA [25–27] or equity-informative cost-effectiveness analysis [1, 10, 13, 66] have been published. This study systematically organizes and synthesizes efficiency-equity trade-off outcomes from DCEA applications in healthcare, and by employing two complementary tools to assess the quality of the included literature, thereby adding a new chapter to the literature.

Another DCEA review found that none of the DCEA studies published before 2023 were conducted in the context of COVID-19. However, by including newly published articles from years of 2024 and 2025, we identified three articles [44, 53, 61] addressing COVID-19. Given that increased public health expenditures during the pandemic aim to improve health outcomes and reduce health inequalities [67], integrating equity considerations into economic evaluation tools such as DCEA can provide valuable insights for policy decision-making, which indicates that policymakers are gradually shifting from the then-prevalent, more traditional CEA approach toward DCEA research [4].

Furthermore, this study introduces a novel methodological analytical framework to systematically examine the key methodological dimensions of DCEA empirical research, specifically, the rationale for subgroup classification, modeling methodology, and the selection of equity indexes. It also examines distinctions between A-DCEA and DCEA, whereas previous systematic reviews primarily summarized basic characteristics and introduced new methods based on the advantages and disadvantages of DCEA analysis workflows, this study provides more systematic methodological evidence for the field.

This systematic review identified 28 relevant DCEA empirical studies. Included studies were published after 2017, particularly after the COVID-19 pandemic, which further increased policymakers’ focus on DCEA. The peak observed in 2025, with nine studies published, can be attributed in part to the typical research cycle, as foundational methodological guidance emerged around 2021 [17]. Nevertheless, the year-to-year fluctuations in publication output suggest that the field remains in its formative stages and has yet to establish a sustained trajectory of research growth, reflecting an ongoing lack of methodological consensus [22].

In terms of scope, research using DCEA to evaluate health policy interventions covered various diseases, intervention types, and populations, spanning low-, middle-, and high-income countries. This suggests that DCEA may have broad application to explore trade-offs and intersections between efficiency and equity in Health Technology Assessments (HTAs) and other decision-making contexts. The concentration of research on infectious diseases and cancers (eight and six studies, respectively) suggests that DCEA has been readily adopted in disease areas with well-understood epidemiology, clear intervention pathways, and established evidence bases. However, research on mental health, rare diseases, or systemic interventions beyond infectious disease control remains relatively scarce. This may stem from data limitations, highlighting conceptual challenges in defining “equity-relevant subgroups” [66].

The research mostly focuses on the health system perspective, possibly related to the greater significance of including equity considerations in public policy making, and the relatively good data accessibility of DCEA studies. While traditional CEA adopts patient and healthcare institution perspectives [68], the absence of these viewpoints in DCEA may reveal a potential limitation: when equity is reduced to macro-level statistical indicators such as the Gini coefficient or Atkinson index, alongside system-level opportunity cost calculations, the complex manifestations of inequality within individual healthcare experiences and micro-level medical practices may be obscured. How patients perceive inequality and how healthcare institutions exacerbate or mitigate it in resource allocation remain questions, which suggests that future research should expand its analytical horizons.

A quality appraisal of the included studies was conducted using the CHEERS 2022 and QHES instruments. The CHEERS 2022 assessment indicated that the reporting of DCEA literature was generally complete; however, no study reported a health economic analysis plan. This absence may be partly attributed to the fact that ten of the included studies were published prior to the release of the CHEERS 2022 statement. Furthermore, the reporting of key items such as time horizon and the effect of engagement with patients and others affected by the study requires strengthening. The QHES assessment revealed that the overall quality of the articles was high, but the specification and justification of the research perspective were often incomplete. Both assessment tools also indicated that several DCEA studies could be improved in terms of reporting discounting rates, the rationale and description of the model, and the handling of uncertainty.

Methodologically, the key to DCEA is the quantitative calculation of health equity and the adjustment of health benefits based on equity [17]. The equity consideration process involves two aspects: First, equity impact analysis: identifying the main factors influencing equity, dividing the overall population’s health output into different subgroups according to the main influencing factors, and calculating the health differences and their equity among the different subgroups [66]. Second, equity weight analysis: using the inequality aversion parameter ε to measure the public’s level of concern about health differences [69].

The selection of the main factors influencing equity directly defines the subgroup division method in the model and fundamentally affects the direction of data collection and parameterization [23]. Current research [12, 70] has found that numerous factors are closely related to health equity, including age, sex, race and ethnicity, geographic location, and a series of socioeconomic attributes that are usually classified as social determinants or social needs of health. Among these, some characteristics (such as age and sex) are objective and easy to assess, while others (such as socioeconomic poverty and race) are more difficult to define due to their conceptual complexity and social constructiveness [71, 72]. Avanceña et al. (2020) [10]. found that existing DCEA research commonly uses subgroup classification variables in (d) Societal and structural and (b) Financial and economic, consistent with our research findings.

However, this study also reveals that even seemingly straightforward categories of factors exhibit heterogeneity during operationalization. Take, for example, the three studies that used geographic regions as their classification basis. Lee et al. (2018) [36] categorized South Korea’s 16 administrative regions based on mortality outcomes, treating geography as a policy target area and implementing interventions solely based on health outcomes without explicitly linking economic or social variables. In contrast, Love-Koh et al. (2020) [41] explicitly linked Brazil’s 27 states to economic variables and state-specific health opportunity costs, where geographic location partially served as a proxy for socioeconomic status, thereby capturing financial and structural preconditions of inequality. Meanwhile, Olsen et al. (2021) [43] introduced an urban-rural dichotomy across Ethiopia’s 11 major administrative regions, deeply binding geography to both social structural variables (urban/rural attributes) and economic variables (intervention costs).

Although current research generally agrees that using the weighting method based on equity considerations is a reasonable research approach [69, 73], there is still no broad consensus on which indicators should be assigned weights and how the weights should be allocated. Some scholars advocate setting weights based on socioeconomic factors in order to more fully reflect the attention to disadvantaged groups in the analysis. However, in the process of selecting weight variables, a trade-off between universality and practicality still needs to be made, because many weight decisions depend on value judgments in specific contexts and may change with differences in social preferences [66]. For example, in the DCEA study in the United States, 90.1% of the subgroup classification dimensions included “race/ethnicity”. Beyond this, concerns related to health inequalities have gradually expanded to encompass multidimensional vulnerable groups, such as the poor (Ethiopia, Malawi) [35, 38, 47, 57], the elderly (the United States) [49], and women (the United States) [58]. Despite the existence of the above social differences, all parties generally agree that people with more severe illnesses should be given higher weights [48]. However, this consensus remains at the conceptual level, lacking unified operational standards and significant disagreements persist regarding the "degree of priority". Furthermore, the quantification of these characteristics faces significant challenges due to the difficulty in obtaining reliable and critical health statistics [74]. Due to weak data foundations, reliance on intermittent surveys, and the lack of systematic registration, making DCEA implementation in LMICs even more challenging [75].

Specifically, suppose policymakers wish to conduct a clear quantitative assessment of health equity issues. In that case, they should clearly define and determine their equity priorities (such as the special focus on race/ethnicity in the US) and propose standards or indicators for identifying equity subgroups and stratifying data. In the UK, a commonly used indicator for measuring relative socioeconomic poverty is the IMD, which covers seven dimensions of poverty and is estimated in small areas of approximately 1,500 people [76]. Many countries have similar composite poverty indices that can be used to understand the impact of equity [77]. Stanimirovic et al. (2025) [51] constructed a candidate variable pool and planned to select the top 5 subgroup classification variables based on importance using the Delphi method. Conversely, epidemiological data could be stratified according to existing relevant equity classifications to define population subgroups in DCEA.

In terms of modeling approaches, Markov models emerged as the most frequently used approach, appearing in seven studies. As a classic cohort model, Markov models simulate the long-term health and economic outcomes of a target population by defining mutually exclusive health states and the probabilities of transitions between states [78]. Their popularity can be attributed to the clear structure and computational efficiency, making them particularly suitable for rapid assessments with limited data [53]. However, Markov models have limitations in handling individual heterogeneity and complex life trajectories [79].

Compared to traditional Markov models, Micro-simulation models are more capable of simulating multiple health states and tracking individual patient histories, offering greater flexibility. This capacity positions them as a particularly promising tool for future DCEA applications, but they require more complex parameterization [80]. Collins et al. (2020) [39] explicitly used a dynamic, stochastic Micro-simulation model to evaluate cardiovascular screening in Liverpool. This model was not a static cohort but a dynamic simulation that projected the entire population of Liverpool forward, allowing for the detailed tracking of individual risk factor trajectories and their differential evolution across socioeconomic groups.

Hybrid approaches—Decision tree-partitioned survival model and Decision tree-Markov models are both used for short-term and long-term two-stage analysis [81]. The decision tree stage can characterize short-term event paths, while the Partition survival model stage can calculate the QALY for each path link’s specific Progression-free survival (PFS) and Overall survival (OS) curves [82], and the Markov stage uses the short-term outcome of the decision tree as the initial state, simulating the long-term evolution of the cohort over several years or even decades through state transitions [78].

DCEA modeling approaches exhibit significant diversity. This stems primarily from the heterogeneity of research questions: transmission dynamics vary across diseases (e.g., infectious vs. chronic conditions), intervention types differ (screening, treatment, vaccination), and policy decision points diverge (resource allocation, pricing compensation). For instance, assessing the equity of influenza vaccines necessitates capturing dynamic transmission between populations, pointing toward dynamic transmission models [83]. Conversely, evaluating the cost-effectiveness of cancer screening makes Markov models more suitable due to their clear depiction of disease natural history [78]. Furthermore, disparities in data availability profoundly influence model selection: studies with access to comprehensive administrative databases and long-term follow-up cohorts can support data-intensive Micro-simulation frameworks. Therefore, the current diversity of modeling approaches does not reflect disorder but rather represents the inevitable outcome of DCEA adapting to different decision-making contexts, data constraints, and research objectives. Currently, DCEA has yet to establish a unified “gold standard” [17]. Researchers can draw inspiration from traditional CEA epidemiology, health economics, and complexity science to achieve interdisciplinary tool adaptation and integrated innovation.

Regarding the choice of which inequality measure to use, this may depend on the availability of data and the context of the decision-making process. For example, simple gap statistics and concentration curves are easier to calculate and more readily understood by non-technical personnel [57]. Different equity indicators, due to their inherent mathematical properties, sensitivity to different parts of the distribution, and the social welfare philosophy they imply, will affect the analysis process, interpretation of results, and policy implications of the DCEA at multiple levels.

First, the emphasis of indicators on “absolute” versus “relative” inequality directly leads to different normative data foundation requirements. Absolute indicators, such as Slope inequality index and Absolute gap index, focus on the absolute gap in health outcomes. Relative indicators, such as Atkinson index and Relative gap index, focus on ratios [84]. In the study by Collins B et al. [42], Slope inequality index was used to measure the absolute difference in health gains from the lowest to the highest income states; Relative inequality index was used to explain the relative gap in health gains, implying proportional equity. Furthermore, using absolute indicators tends to lead to a gradual increase in calculated inequality due to inconsistencies in units, influenced by economic development. When the units change, although the actual distribution remains the same, the calculated equity changes. Therefore, relative indicators are more advantageous for measuring equity [15, 17].

Secondly, different indicators show significant differences in their sensitivity to changes in health distribution, and the response functions of various indicators to changes in subgroup population size and extreme values at the tail end of the distribution are different. The Theil index, as a member of the generalized entropy index family, is more sensitive to changes in the low end of the health distribution (i.e., the most vulnerable group) [85]. The Gini coefficient, on the other hand, is more sensitive to population changes in the middle part of the distribution [43]. This difference in sensitivity is not a technical detail, but rather a value judgment about which inequality is more worthy of attention [86].

Finally, the connection between indicators and SWFs improves the integration efficiency and standardization of the DCEA framework. The integration of inequality measures with SWFs (exemplified by the Atkinson indexes and Kolm indexes)—enhances the normative coherence of DCEA by operationalizing societal preferences through inequality aversion parameters (ε and α) [87]. The Atkinson index, in particular, was the most frequently employed equity measure in the included studies, appearing in 20 studies. In contrast, the Gini coefficient and scenario comparison method adopted by Olsen et al. [43] could intuitively show the tension between maximizing health and reducing inequality, but it failed to provide a welfare economics framework that integrated the two into a single decision benchmark. Therefore, adopting indicators related to the welfare function is conducive to enabling DCEA to have the potential to have a normative decision support system with a solid ethical foundation. It is worth noting that the social welfare function needs to take into account many complex factors, including the health status of different groups, different views on equity, and various social values. To construct a function that can accurately reflect the entire society’s preference for the trade-off between equity and efficiency involves a large number of theoretical and empirical studies, the process is challenging, and there is a lack of unified and widely accepted derivation methods and standards.

Notably, six studies [40–42, 47, 51, 60] employed multiple equity indexes concurrently, suggesting that researchers recognized the value of triangulating different equity indexes. Quan et al. (2021) [42] used both Theil index and Difference index. The former believed that issues affecting larger groups had higher social priority; the latter insisted that regardless of group size, the injustices suffered should receive equal moral weight [88].

The finding that the majority of included studies (85.71%) reported win-win outcomes aligns with previous research [63]. Only one study [48] reported a lose-lose outcome: the Prevention of overweight in infancy (POI) -Combo childhood obesity intervention in Australia. Its high implementation costs resulted in a net health loss, and its effects disproportionately benefited higher socioeconomic groups, ultimately widening rather than reducing inequalities. Other researchers found that high-cost hospital specialist treatment, due to its resource intensity and main benefit to the privileged groups, often falls into the third quadrant [75].

Trade-offs between equity and efficiency are particularly prominent in the field of preventive medicine, as exemplified by the liquid biopsy-first strategy for lung cancer in the United States [60], cervical cancer screening strategies in Uganda [34], and the pro-poor rotavirus vaccination programme in Ethiopia [35]. Taking Uganda’s cervical cancer screening strategy as an example, under low baseline coverage (30%), expanding coverage (one lifetime screening) falls within the first quadrant. However, increasing screening frequency (two to three lifetime screenings) while boosting total health outcomes exacerbated health disparities between screened and unscreened populations, placing it in the second quadrant.

It is worth noting that A-DCEA, as a more simplified alternative to DCEA, should have distinct application scenarios. These depend on data availability, decision-making needs, and resource constraints [89]. DCEA is appropriate in contexts where comprehensive subgroup data (including baseline health, healthcare utilization, intervention effects, and opportunity costs) are accessible. This makes it particularly suitable for evaluating complex, long-term public health interventions [35, 37], chronic disease management programs [39, 57], or screening initiatives with multi-dimensional equity considerations. In contrast, A-DCEA offers a pragmatic alternative when only aggregate data (i.e., average treatment effects, target population size, and broad patterns of healthcare use) are available. It is especially valuable for rapid decision-making, preliminary intervention screening, or evaluations in resource-limited settings, including low-income regions [45] or during public health emergencies [56], where detailed subgroup data are difficult or costly to obtain.

### Limitations

While our review synthesized evidence across multiple studies, several important limitations should be acknowledged. First, we searched multiple databases to enhance the comprehensiveness of our retrieval, but articles included in other databases and those in the process of publication may still have been missed. Therefore, biases associated with our search strategy couldn't be entirely ruled out. Second, by excluding conference abstracts, our review might have inadvertently omitted studies reporting interim results or preliminary findings from ongoing research. Such studies could offer valuable early insights into emerging DCEA methodologies. Third, although the CHEERS 2022 and QHES included items related to equity—such as distribution effects and differences in outcomes across subgroups, these scales didn't not explicitly focus on DCEA, but only on the reporting method. This indicates the need to develop a dedicated quality assessment tool for DCEA studies. Moreover, the application of the QHES tool relied on binary judgments of “yes” or “no” for each entry, lacking intermediate metric values on a continuous scale. This approach resulted in the loss of important information in practical applications. Fourth, the data extraction form was not formally piloted prior to its use. While we developed the checklist based on three rounds of team discussion, the absence of formal piloting may have introduced inconsistencies or omissions in data extraction.

## Conclusions

As an important tool for promoting equitable health decisions, DCEA still faces challenges in its routine implementation and consistent application. This study systematically reviews three key aspects of DCEA: the basis for subgroup classification, different modeling methodologies and equity measurement indexes. It also synthesizes the efficiency-equity trade-off outcomes of DCEA. The findings reveal three core obstacles to DCEA development: methodologically, a lack of universally accepted standards and measurement frameworks for equity subgrouping; data-wise, difficulties in obtaining evidence for key equity parameters; and cognitively, decision-makers’ relative unfamiliarity with the methodology and the lack of a clear equity priority. Notably, DCEA retains inherent limitations of traditional CEA, particularly its inability to capture differences in healthcare service quality and welfare coverage, limiting its comprehensive reflection of real-world inequalities.

To promote the standardized development of DCEA and enhance its decision-support value, the following recommendations are made. First, advance the standardization process. It is suggested that HTA agencies and government departments jointly lead the establishment of a standardized framework (quality assessment tool) for DCEA application, clearly defining measurement content and methods to enhance the comparability, robustness, and decision relevance of research results. Second, construct an ecosystem of equity evidence. The data foundation should be systematically improved through the following pathways: promoting cross-domain database links and integrating equity-related variables; enriching existing registration systems and Real-world data (RWD) resources; supporting observational and longitudinal study designs that incorporate DCEA dimensions; and particularly strengthening the development of health information systems in LMICs, in accordance with WHO standards. Finally, multi-stakeholder collaboration should be strengthened. It is recommended to use qualitative research methods such as in-depth interviews and focus groups to gain a deeper understanding of the practical obstacles and improvement pathways for DCEA application in specific contexts. Furthermore, future research should pay particular attention to the unique application characteristics of DCEA in different resource environments, which will help reveal challenges not fully covered in this study and promote the establishment of a more inclusive and practical health equity assessment system.

## Supplementary Information

Supplementary Material 1.

## Data Availability

The datasets generated and/or analyzed during the current study are available from the corresponding author on reasonable request.

## Abbreviations

DCEA: Distributional cost-effectiveness analysis
QHES: Quality of Health Economic Studies
CHEERS: Consolidated Health Economic Evaluation Reporting Standards
A-DCEA: Aggregate distributional cost-effectiveness analysis
WHO: World Health Organization
COVID-19: Coronavirus Disease 2019
CEA: Cost-effectiveness analysis
ISPOR: International Society for Pharmacoeconomics and Outcomes Research
LMICs: Low-and middle-income countries
EWM: Equity-based weighting method
ECEA: Extended cost-effectiveness analysis
MCDA: Multi-criteria decision analysis
SWFs: Social welfare functions
EDEH: Equally distributed equivalent health
PRISMA: Preferred Reporting Items for Systematic reviews and Meta-Analysis
HIV: Human immunodeficiency virus
iNHB: incremental Net health benefit
iEDEH: increment Equally distributed equivalent health
ICER: Incremental cost-effectiveness ratio
IMD: Index of Multiple Deprivation
SVI: Social Vulnerability Index
SEP/SES: Socioeconomic position /status
HWQ: Household Wealth Quintile
IWI: International Wealth Index
MMs: Micro-simulation models
SEP-MMs: Socioeconomic status-specific micro-simulation models
MCMC-MMs: Monte Carlo Markov-chain micro-simulation models
DSM: Discrete Individual Simulation Model
QALY: Quality-adjusted life year
HTAs: Health Technology Assessments
PFS: Progression-free survival
OS: Overall survival
RWD: Real-world data
