# Supplementary material for: Methodological heterogeneity and equity challenges of distributional cost-effectiveness analysis in healthcare: a systematic review from 2017 to 2025
Source: Arch Public Health. 2026 Apr 16;84:121. doi: 10.1186/s13690-026-01908-0 (PMC13214177; doi:10.1186/s13690-026-01908-0)
Supplement: Supplementary file 1 — Supplementary Material 1. [file 13690_2026_1908_MOESM1_ESM.docx]

**Table 1.** Preferred Reporting Items for Systematic reviews and Meta-Analyses (PRISMA) 2020 checklist for the systematic review of distributional cost-effectiveness analysis studies in healthcare published from inception to 30th August 2025.

| **Section and Topic** | **Item #** | **Checklist item** | **Location where item is reported** |
| --- | --- | --- | --- |
| **TITLE** | | |  |
| Title | 1 | Identify the report as a systematic review. | Front page |
| **ABSTRACT** | | |  |
| Abstract | 2 | See the PRISMA 2020 for Abstracts checklist. | Abstract |
| **INTRODUCTION** | | |  |
| Rationale | 3 | Describe the rationale for the review in the context of existing knowledge. | Introduction |
| Objectives | 4 | Provide an explicit statement of the objective(s) or question(s) the review addresses. | Introduction |
| **METHODS** | | |  |
| Eligibility criteria | 5 | Specify the inclusion and exclusion criteria for the review and how studies were grouped for the syntheses. | Methods |
| Information sources | 6 | Specify all databases, registers, websites, organisations, reference lists and other sources searched or consulted to identify studies. Specify the date when each source was last searched or consulted. | Methods |
| Search strategy | 7 | Present the full search strategies for all databases, registers and websites, including any filters and limits used. | Methods |
| Selection process | 8 | Specify the methods used to decide whether a study met the inclusion criteria of the review, including how many reviewers screened each record and each report retrieved, whether they worked independently, and if applicable, details of automation tools used in the process. | Methods |
| Data collection process | 9 | Specify the methods used to collect data from reports, including how many reviewers collected data from each report, whether they worked independently, any processes for obtaining or confirming data from study investigators, and if applicable, details of automation tools used in the process. | Methods |
| Data items | 10a | List and define all outcomes for which data were sought. Specify whether all results that were compatible with each outcome domain in each study were sought (e.g. for all measures, time points, analyses), and if not, the methods used to decide which results to collect. | Methods |
|  | 10b | List and define all other variables for which data were sought (e.g. participant and intervention characteristics, funding sources). Describe any assumptions made about any missing or unclear information. | Methods |
| Study risk of bias assessment | 11 | Specify the methods used to assess risk of bias in the included studies, including details of the tool(s) used, how many reviewers assessed each study and whether they worked independently, and if applicable, details of automation tools used in the process. | Methods |
| Effect measures | 12 | Specify for each outcome the effect measure(s) (e.g. risk ratio, mean difference) used in the synthesis or presentation of results. | NA |
| Synthesis methods | 13a | Describe the processes used to decide which studies were eligible for each synthesis (e.g. tabulating the study intervention characteristics and comparing against the planned groups for each synthesis (item #5)). | NA |
|  | 13b | Describe any methods required to prepare the data for presentation or synthesis, such as handling of missing summary statistics, or data conversions. | NA |
|  | 13c | Describe any methods used to tabulate or visually display results of individual studies and syntheses. | Methods |
|  | 13d | Describe any methods used to synthesize results and provide a rationale for the choice(s). If meta-analysis was performed, describe the model(s), method(s) to identify the presence and extent of statistical heterogeneity, and software package(s) used. | NA |
|  | 13e | Describe any methods used to explore possible causes of heterogeneity among study results (e.g. subgroup analysis, meta-regression). | NA |
|  | 13f | Describe any sensitivity analyses conducted to assess robustness of the synthesized results. | NA |
| Reporting bias assessment | 14 | Describe any methods used to assess risk of bias due to missing results in a synthesis (arising from reporting biases). | Methods |
| Certainty assessment | 15 | Describe any methods used to assess certainty (or confidence) in the body of evidence for an outcome. | Methods |
| **RESULTS** | | |  |
| Study selection | 16a | Describe the results of the search and selection process, from the number of records identified in the search to the number of studies included in the review, ideally using a flow diagram. | Results |
|  | 16b | Cite studies that might appear to meet the inclusion criteria, but which were excluded, and explain why they were excluded. | Results |
| Study characteristics | 17 | Cite each included study and present its characteristics. | Results |
| Risk of bias in studies | 18 | Present assessments of risk of bias for each included study. | NA |
| Results of individual studies | 19 | For all outcomes, present, for each study: (a) summary statistics for each group (where appropriate) and (b) an effect estimate and its precision (e.g. confidence/credible interval), ideally using structured tables or plots. | NA |
| Results of syntheses | 20a | For each synthesis, briefly summarise the characteristics and risk of bias among contributing studies. | Supplementary material 1 |
|  | 20b | Present results of all statistical syntheses conducted. If meta-analysis was done, present for each the summary estimate and its precision (e.g. confidence/credible interval) and measures of statistical heterogeneity. If comparing groups, describe the direction of the effect. | NA |
|  | 20c | Present results of all investigations of possible causes of heterogeneity among study results. | NA |
|  | 20d | Present results of all sensitivity analyses conducted to assess the robustness of the synthesized results. | NA |
| Reporting biases | 21 | Present assessments of risk of bias due to missing results (arising from reporting biases) for each synthesis assessed. | NA |
| Certainty of evidence | 22 | Present assessments of certainty (or confidence) in the body of evidence for each outcome assessed. | NA |
| **DISCUSSION** | | |  |
| Discussion | 23a | Provide a general interpretation of the results in the context of other evidence. | Discussion |
|  | 23b | Discuss any limitations of the evidence included in the review. | Limitations |
|  | 23c | Discuss any limitations of the review processes used. | Limitations |
|  | 23d | Discuss implications of the results for practice, policy, and future research. | Conclusion |
| **OTHER INFORMATION** | | |  |
| Registration and protocol | 24a | Provide registration information for the review, including register name and registration number, or state that the review was not registered. | Methods |
|  | 24b | Indicate where the review protocol can be accessed, or state that a protocol was not prepared. | Methods |
|  | 24c | Describe and explain any amendments to information provided at registration or in the protocol. | Methods |
| Support | 25 | Describe sources of financial or non-financial support for the review, and the role of the funders or sponsors in the review. | Declaration |
| Competing interests | 26 | Declare any competing interests of review authors. | Declaration |
| Availability of data, code and other materials | 27 | Report which of the following are publicly available and where they can be found: template data collection forms; data extracted from included studies; data used for all analyses; analytic code; any other materials used in the review. | NA |

*Note:* Page MJ, McKenzie JE, Bossuyt PM, Boutron I, Hoffmann TC, Mulrow CD, et al. The PRISMA 2020 statement: an updated guideline for reporting systematic reviews. BMJ 2021;372:n71. doi: 10.1136/bmj.n71. This work is licensed under CC BY 4.0. To view a copy of this license, visit <https://creativecommons.org/licenses/by/4.0/>

**Table 2.** Search strategies and number of records retrieved from each database for the systematic review of distributional cost-effectiveness analysis studies in healthcare published from inception to 30th August 2025.

| **Retrieval Database** | **Item** | **Search query** |
| --- | --- | --- |
| web of science | Total | ((To= (Distributional cost effectiveness analysis) OR (DCEA) OR (distributional economic evaluation) AND AB=(equity) OR (inequality) OR (fairness) OR (opportunity cost) |
|  | #1 | 【Topic】(Distributional cost effectiveness analysis) OR (DCEA) OR (distributional economic evaluation) |
|  | #2 | 【Abstract】(equity) OR (inequality) OR (fairness) OR (opportunity cost) |
|  | Result | 252 |
| PubMed | Total | ((Distributional cost effectiveness analysis [Title/Abstract]) OR (DCEA[Title/Abstract]) OR (distributional economic evaluation [Title/Abstract]) OR (health distribution [Title/Abstract])) AND ((economic evaluation) OR (equity) OR (inequality) OR (opportunity cost) OR (fairness)) |
|  | #1 | (Distributional cost effectiveness analysis [Title/Abstract]) OR (DCEA[Title/Abstract]) OR (distributional economic evaluation [Title/Abstract]) OR (health distribution [Title/Abstract]) |
|  | #2 | (economic evaluation) OR (equity) OR (inequality) OR (opportunity cost) |
|  | Result | 158 |
| the Cochrane Library | Total | Title Abstract Keyword= (Distributional cost effectiveness analysis) OR (DCEA) OR (distributional economic evaluation) OR (health distribution)  AND Title Abstract Keyword= (health distribution) AND ((equity) OR (inequality) OR (fairness) OR (opportunity cost) |
|  | #1 | Title Abstract Keyword= (Distributional cost effectiveness analysis) OR (DCEA) OR (distributional economic evaluation) OR (health distribution) |
|  | #2 | Title Abstract Keyword= (health distribution) AND ((equity) OR (inequality) OR (fairness) OR (opportunity cost) |
|  | Result | 452 |
| CNKI | Total | 【Title/Keywords/Abstract】(Precise) Distributional Cost-Effectiveness Evaluation + Distributional Cost-Effectiveness Analysis + DCEA |
|  | Result | 25 |
| Wangfang | Total | 【Title or Keywords】(Vague) "Distributional Cost-Effectiveness Evaluation" OR "Distributional Cost-Effectiveness Analysis" OR “DCEA” |
|  | Result | 8 |
| VIP | Total | 【Title or Keywords】(Vague)"Distributional Cost-Effectiveness Evaluation" + "Distributional Cost-Effectiveness Analysis" + “DCEA” |
|  | Result | 28 |

**Table 3.** Consolidated Health Economic Evaluation Reporting Standards (CHEERS) 2022 checklist items for assessing the reporting quality of the 28 included distributional cost-effectiveness analysis studies published from 2017 to 2025.

| **Section/topic** | **Item no.** | **Guidance for reporting** |
| --- | --- | --- |
| Title | 1 | Identify the study as an economic evaluation and specify the interventions being compared. |
| Abstract | 2 | rovide a structured summary that highlights context, key methods, results, and alternative analyses. |
| **Introduction** | | |
| Background and  objectives | 3 | Give the context for the study, the study question, and its practical relevance for decision making in policy or practice. |
| **Methods** | | |
| Health economic analysis plan | 4 | Indicate whether a health economic analysis plan was developed and where available. |
| Study population | 5 | Describe characteristics of the study population (such as age range, demographics, socioeconomic, or clinical characteristics). |
| Setting and location | 6 | Provide relevant contextual information that may influence findings. |
| Comparators | 7 | Describe the interventions or strategies being compared and why chosen. |
| Perspective | 8 | State the perspective(s) adopted by the study and why chosen. |
| Time horizon | 9 | State the time horizon for the study and why appropriate. |
| Discount rate | 10 | Report the discount rate(s) and reason chosen. |
| Selection of outcomes | 11 | Describe what outcomes were used as the measure(s) of benefit(s) and harm(s). |
| Measurement of outcomes | 12 | Describe how outcomes used to capture benefit(s) and harm(s) were measured. |
| Valuation of outcomes | 13 | Describe the population and methods used to measure and value outcomes. |
| Measurement and valuation of resources and costs | 14 | Describe how costs were valued. |
| Currency, price date, and conversion | 15 | Report the dates of the estimated resource quantities and unit costs, plus the currency and year of conversion. |
| Rationale and description of model | 16 | If modeling is used, describe in detail and why used. Report if the model is publicly available and where it can be accessed. |
| Analytics and assumptions | 17 | Describe any methods for analysing or statistically transforming data, any extrapolation methods, and approaches for validating any model used. |
| Characterizing heterogeneity | 18 | Describe any methods used for estimating how the results of the study vary for subgroups. |
| Characterizing distributional effects | 19 | Describe how impacts are distributed across different individuals or adjustments made to reflect priority populations. |
| Characterizing uncertainty | 20 | Describe methods to characterize any sources of uncertainty in the analysis. |
| Approach to engagement with patients and others affected by the study | 21 | Describe any approaches to engage patients or service recipients, the general public, communities, or stakeholders (such as clinicians or payers) in the design of the study. |
| **Results** | | |
| Study parameters | 22 | Report all analytic inputs (such as values, ranges, references) including uncertainty or distributional assumptions. |
| Summary of main results | 23 | Report the mean values for the main categories of costs and outcomes of interest and summarize them in the most appropriate overall measure. |
| Effect of uncertainty | 24 | Describe how uncertainty about analytic judgments, inputs, or projections affect findings. Report the effect of choice of discount rate and time horizon, if applicable. |
| **Discussion** | | |
| Effect of engagement with patients and others affected by the study | 25 | Report on any difference patient/service recipient, general public, community, or stakeholder involvement made to the approach or findings of the study |
| Study findings, limitations, generalizability, and current knowledge | 26 | Report key findings, limitations, ethical or equity considerations not captured, and how these could affect patients, policy, or practice. |
| Source of funding | 27 | Describe how the study was funded and any role of the funder in the identification, design, conduct, and reporting of the analysis |
| Conflicts of interest | 28 | Report authors conflicts of interest according to journal or International Committee of Medical Journal Editors requirements. |

**Table 4.** Detailed Consolidated Health Economic Evaluation Reporting Standards (CHEERS) 2022 quality evaluation results for the 28 included distributional cost-effectiveness analysis studies published from 2017 to 2025.

| No. | Author | Campos, et al. | Lee, et al. | Dawkins, et al. | Chang, et al. | Arnold, et al. | Collins, et al. | Love-Koh, et al. | Love-Koh, et al. | Quan , et al. | Olsen, et al. | Kowal, et al. | Meunier , et al. | Goshua, et al. | Assebe, et al. |
| --- | --- | --- | --- | --- | --- | --- | --- | --- | --- | --- | --- | --- | --- | --- | --- |
|  | Publication Year | 2017 | 2018 | 2018 | 2018 | 2020 | 2020 | 2020 | 2021 | 2021 | 2021 | 2022 | 2023 | 2023 | 2023 |
| 1 | Title | Title | Title | Title | Title | Title | Title | Title | Title | Title | Title | Title | Title | Title | Title |
| 2 | Abstract | Abstract | Abstract | Abstract | Abstract | Abstract | Abstract | Abstract | Abstract | Abstract | Abstract | Abstract | Abstract | Abstract | Abstract |
| 3 | Background and  objectives | Introduction | Introduction | Introduction | Introduction | Introduction | Introduction | Introduction | Introduction | Introduction | Introduction | Introduction | Introduction | Introduction | Introduction |
| 4 | Health economic analysis plan | / | / | / | / | / | / | / | / | / | / | / | / | / | / |
| 5 | Study population | Method | Method | Method | Method | Method | Method | Method | Method | Method | Method | Method | Method | Method | Method |
| 6 | Setting and location | Introduction、  Method | Introduction、  Method | Method | Introduction、  Method | Method | Introduction、  Method | Introduction、  Method | Introduction、  Method | Introduction、  Method | Introduction、  Method | Introduction、  Method | Introduction、  Method | Method | Introduction、  Method |
| 7 | Comparators | Method | Method | Method | Method | Method | Method | Method | Method | Method | Method | Method | Method | Method | Method |
| 8 | Perspective | Method | Method | Method | Method | Method | Method | Method | Method | Method | Method | Method | Method | Method | Method |
| 9 | Time horizon | Method | / | / | Method | / | Method | Method | Method | Method | Method | / | / | Method | Method |
| 10 | Discount rate | Method | Method | / | / | / | Method | Method | / | Method | Method | / | / | Method | / |
| 11 | Selection of outcomes | Method | Method，Results | Method | Method，Results | Method | Method，Results | Method | Method、Results | Method，Results | Method，Results | Method、Results | Method、Results | Method | Method，Results |
| 12 | Measurement of outcomes | Method | Method、Results | Method | Method、Results | Method | Method、Results | Method | Method、Results | Method、Results | Method、Results | Method、Results | Method、Results | Method | Method、Results |
| 13 | Valuation of outcomes | Method | Method | Method | Method | Method | Method | Method | Method、  Results | Method | Method | Method | Method | Method | Method |
| 14 | Measurement and valuation of resources and costs | Method | Method | Method | Method | Method | Method | Method | Method | Method | Method | Method | Method | Method | Method |
| 15 | Currency, price date, and conversion | Method | Method | Method | Method | Method | Method | Method | Method | Method | Method | / | / | Method | Method |
| 16 | Rationale and description of model | Method | Method | / | Method | / | Method | Method | Method | Method | Method | Method | Method | Method | Method |
| 17 | Analytics and assumptions | Method | Method | Method | Method | Method | Method | Method | Method | Method | Method | Method | Method | Method | Method |
| 18 | Characterizing heterogeneity | Method | Method | Method | Method | Method | Method | Method | Method | Method | Method | Method | Method | Method | Method |
| 19 | Characterizing distributional effects | Method | Method，Results | Method | Method，Results | Method | Method，Results | Method | Method | Method | Method，Results | Method、Results | Method、Results | Method | Method，Results |
| 20 | Characterizing uncertainty | Method | / | Method | Method | Method | Method | Method | Method | Method | / | Method、  Results | Method、  Results | Method | Method |
| 21 | Approach to engagement with patients and others affected by the study | / | / | / | / | / | / | / | / | / | / | / | / | / | / |
| 22 | Study parameters | Method、Results | Method、Results | Method、Results | Method、Results | Method、Results | Method、Results | Method、Results | Method、Results | Method、Results | Method、Results | Method、Results | Method、Results | Method、Results | Method、Results |
| 23 | Summary of main results | Results | Results | Results | Results | Results | Results | Results | Results | Results | Results | Results | Results | Results | Results |
| 24 | Effect of uncertainty | Method、Results | Method、Results | Method、Results | Method、Results | Method、Results | Method、Results | Method、Results | Method、Results | Method、Results | Method、Results | Method、Results | Method、Results | Method、Results | Method、Results |
| 25 | Effect of engagement with patients and others affected by the study | / | / | / | / | / | / | / | / | / | / | / | / | / | / |
| 26 | Study findings, limitations, generalizability, and current knowledge | Discussion | Discussion | Discussion | Discussion | Discussion | Discussion | Discussion | Discussion | Discussion | Discussion | Discussion | Discussion | Discussion | Discussion |
| 27 | Source of funding | Declaration | Declaration | Declaration | Declaration | Declaration | Declaration | Declaration | Declaration | Declaration | Declaration | Declaration | Declaration | Declaration | Declaration |
| 28 | Conflicts of interest | Declaration | Declaration | Declaration | Declaration | Declaration | Declaration | Declaration | Declaration | Declaration | Declaration | Declaration | Declaration | Declaration | Declaration |

**Table 4.** Continuation Table

| No. | Author | Killedar, et al. | Kwon, et al. | Meunier, et al. | Stanimirovic, et al. | Synnott , et al. | Kowal, et al. | Biundo, et al. | Majda, et al. | Walbaum, et al. | Barry , et al. | Tschampl, et al. | Jansen, et al. | Jansen, et al. | Okafor, et al. |
| --- | --- | --- | --- | --- | --- | --- | --- | --- | --- | --- | --- | --- | --- | --- | --- |
|  | Publication Year | 2023 | 2023 | 2024 | 2024 | 2024 | 2024 | 2024 | 2025 | 2025 | 2025 | 2025 | 2025 | 2025 | 2025 |
| 1 | Title | Title | Title | Title | Title | Title | Title | Title | Title | Title | Title | Title | Title | Title | Title |
| 2 | Abstract | Abstract | Abstract | Abstract | Abstract | Abstract | Abstract | Abstract | Abstract | Abstract | Abstract | Abstract | Abstract | Abstract | Abstract |
| 3 | Background and  objectives | Introduction | Introduction | Introduction | Introduction | Introduction | Introduction | Introduction | Introduction | Introduction | Introduction | Introduction | Introduction | Introduction | Introduction |
| 4 | Health economic analysis plan | / | / | / | / | / | / | / | / | / | / | / | / | / | / |
| 5 | Study population | Method | Method | Method | Method | Method | Method | Method | Method | Method | Method | Method | Method | Method | Method |
| 6 | Setting and location | Introduction、  Method | Introduction、  Method | Method | Introduction、  Method | Introduction、  Method | Introduction、  Method | Introduction、  Method | Introduction、  Method | Introduction、  Method | Introduction、  Method | Introduction、  Method | Introduction、  Method | Introduction、  Method | Introduction、  Method |
| 7 | Comparators | Method | Method | Method | Method | Method | Method | Method | Method | Method | Method | Method | Method | Method | Method |
| 8 | Perspective | Method | Method | Method | Method | Method | Method | Method | Method | Method | Method | Method | Method | Method | Method |
| 9 | Time horizon | Method | Method | Method | / | Method | Method | Method | Method | Method | Method | Method | Method | Method | Method |
| 10 | Discount rate | Method | Method | / | / | Method | Method | Method | Method | / | Method | Method | Method | Method | Method |
| 11 | Selection of outcomes | Method，Results | Method | Method、Results | Method | Method | Method，Results | Method | Method、Results | Method，Results | Method | Method，Results | Method，Results | Method，Results | Method，Results |
| 12 | Measurement of outcomes | Method、Results | Method | Method | Method | Method | Method、Results | Method | Method | Method、Results | Method | Method、Results | Method、Results | Method、Results | Methd、  Results |
| 13 | Valuation of outcomes | Method | Method | Method | Method | Method | Method | Method | Method | Method | Method | Method | Method | Method | Method |
| 14 | Measurement and valuation of resources and costs | Method | Method | Method | Method | Method | Method | Method | Method | Method | Method | Method | Method | Method | Method |
| 15 | Currency, price date, and conversion | Method | Method | Method | / | / | Method | Method | Method | Method | Method | Method | Method | Method | Method |
| 16 | Rationale and description of model | Method | Method | Method | Method | Method | Method | Method | Method | Method | Method | Method | Method | Method | Method |
| 17 | Analytics and assumptions | Method | Method | Method | Method | Method | Method | Method | Method | Method | Method | Method | Method | Method | Method |
| 18 | Characterizing heterogeneity | Method | Method | Method | Method | Method | Method | Method | Method | Method | Method | Method | Method | Method | Method |
| 19 | Characterizing distributional effects | Method，Results | Method | Method | Method | Method，Results | Method | Method，Results | Method | Method，Results | Method | Method，Results | Method，Results | Method | Method，Results |
| 20 | Characterizing uncertainty | Method | Method | Method | Method | Method | Method | Method | Method、Results | Method | Method | Method | Method | Method | Method |
| 21 | Approach to engagement with patients and others affected by the study | / | / | / | Method | / | / | / | / | / | / | / | / | / | / |
| 22 | Study parameters | Method、Results | Method、Results | Method、Results | Method、Results | Method、Results | Method、Results | Method、Results | Method、Results | Method、Results | Method、Results | Method、Results | Method、Results | Method、Results | Method、Results |
| 23 | Summary of main results | Results | Results | Results | Results | Results | Results | Results | Results | Results | Results | Results | Results | Results | Results |
| 24 | Effect of uncertainty | Method、Results | Method、Results | Method、Results | Method、Results | Method、Results | Method、Results | Method、Results | Method、Results | Method、Results | Method、Results | Method、Results | Method、Results | Method、Results | Method、Results |
| 25 | Effect of engagement with patients and others affected by the study | / | / | / | / | / | / | / | / | / | / | / | / | / | / |
| 26 | Study findings, limitations, generalizability, and current knowledge | Discussion | Discussion | Discussion | Discussion | Discussion | Discussion | Discussion | Discussion | Discussion | Discussion | Discussion | Discussion | Discussion | Discussion |
| 27 | Source of funding | Declaration | Declaration | Declaration | Declaration | Declaration | Declaration | Declaration | Declaration | Declaration | Declaration | Declaration | Declaration | Declaration | Declaration |
| 28 | Conflicts of interest | Declaration | Declaration | Declaration | Declaration | Declaration | Declaration | Declaration | Declaration | Declaration | Declaration | Declaration | Declaration | Declaration | Declaration |

**Table 5.** The Quality of Health Economic Studies (QHES) instrument used for assessing the methodological quality of the 28 included distributional cost-effectiveness analysis studies published from 2017 to 2025.

| **Item** | **Question** | **Point** |
| --- | --- | --- |
| 1 | Was the study objective presented in a clear, specific, and measurable manner? | 7 |
| 2 | Were the perspective of the analysis (societal, third-party payer, etc.) and reasons for its selection stated? | 4 |
| 3 | Were variable estimates used in the analysis from the best available source (i.e., Randomized Control Trial — Best, Expert Opinion — Worst)? | 8 |
| 4 | If estimates came from a subgroup analysis, were the groups prespecified at the beginning of the study? | 1 |
| 5 | Was uncertainty handled by: 1) statistical analysis to address random events; 2) sensitivity analysis to cover a range of assumptions? | 9 |
| 6 | Was incremental analysis performed between alternatives for resources and costs? | 6 |
| 7 | Was the methodology for data abstraction (including the value of health states and other benefits) stated? | 5 |
| 8 | Did the analytic horizon allow time for all relevant and important outcomes? Were benefits and costs that went beyond 1 year discounted (3%–5%) and justification given for the discount rate? | 7 |
| 9 | Was the measurement of costs appropriate and the methodology for the estimation of quantities and unit costs clearly described? | 8 |
| 10 | Were the primary outcome measure(s) for the economic evaluation clearly stated and were the major short-term, long-term, and negative outcomes included? | 6 |
| 11 | Were the health outcomes measures/scales valid and reliable? If previous lytested valid and reliable measures were not available, was justification given for the measures/scales used? | 7 |
| 12 | Were the economic model (including structure), study methods and analysis, and the components of the numerator and denominator displayed in a clear transparent manner? | 8 |
| 13 | Were the choice of economic model, main assumptions and limitations of the study stated and justified? | 7 |
| 14 | Did the author(s) explicitly discuss direction and magnitude of potential biases? | 6 |
| 15 | Were the conclusions/recommendations of the study justified and based on the study results? | 8 |
| 16 | Was there a statement disclosing the source of funding for the study? | 3 |
| **Total Points** |  | **100** |

**Table 6.** Individual Quality of Health Economic Studies (QHES) instrument scores for the 28 included distributional cost-effectiveness analysis studies published from 2017 to 2025.

|  |  | **QHES Question** | | | | | | | | | | | | | | | | | |
| --- | --- | --- | --- | --- | --- | --- | --- | --- | --- | --- | --- | --- | --- | --- | --- | --- | --- | --- | --- |
| **Lead Author** | **Year** | **1** | **2** | **3** | **4** | **5** | **6** | **7** | **8** | **9** | **10** | **11** | **12** | **13** | **14** | **15** | **16** | **Total** |  |
| Campos, et al. | 2017 | 7 | 0 | 8 | 1 | 9 | 6 | 5 | 7 | 8 | 6 | 7 | 8 | 7 | 6 | 8 | 3 | **96** |  |
| Dawkins, et al. | 2018 | 7 | 0 | 8 | 1 | 9 | 6 | 5 | 7 | 8 | 6 | 7 | 8 | 0 | 6 | 8 | 3 | **89** |  |
| Lee, et al. | 2018 | 7 | 4 | 8 | 1 | 0 | 6 | 5 | 0 | 8 | 6 | 7 | 0 | 7 | 6 | 8 | 3 | **76** |  |
| Chang, et al. | 2018 | 7 | 0 | 8 | 1 | 9 | 0 | 5 | 0 | 8 | 6 | 7 | 8 | 7 | 6 | 8 | 3 | **83** |  |
| Arnold, et al. | 2020 | 7 | 0 | 8 | 1 | 9 | 6 | 5 | 0 | 8 | 6 | 7 | 0 | 7 | 6 | 8 | 3 | **81** |  |
| Collins, et al. | 2020 | 7 | 4 | 8 | 1 | 9 | 6 | 5 | 7 | 8 | 6 | 7 | 8 | 7 | 6 | 8 | 3 | **100** |  |
| Love-Koh, et al. | 2020 | 7 | 4 | 8 | 1 | 9 | 6 | 5 | 7 | 8 | 6 | 7 | 8 | 7 | 6 | 8 | 3 | **100** |  |
| Love-Koh, et al. | 2021 | 7 | 0 | 8 | 1 | 9 | 6 | 5 | 0 | 8 | 6 | 7 | 8 | 7 | 6 | 8 | 3 | **89** |  |
| Quan, et al. | 2021 | 7 | 4 | 8 | 1 | 9 | 6 | 5 | 7 | 8 | 6 | 7 | 8 | 7 | 6 | 8 | 3 | **100** |  |
| Olsen, et al. | 2021 | 7 | 0 | 8 | 1 | 0 | 6 | 5 | 7 | 8 | 6 | 7 | 8 | 7 | 6 | 8 | 3 | **87** |  |
| Kowal, et al. | 2023 | 7 | 4 | 8 | 1 | 9 | 6 | 5 | 0 | 8 | 6 | 7 | 8 | 7 | 6 | 8 | 3 | **93** |  |
| Meunier, et al. | 2023 | 7 | 4 | 8 | 1 | 9 | 6 | 5 | 0 | 8 | 6 | 7 | 8 | 7 | 6 | 8 | 3 | **93** |  |
| Goshua, et al. | 2023 | 7 | 4 | 8 | 1 | 9 | 6 | 5 | 7 | 8 | 6 | 7 | 8 | 7 | 6 | 8 | 3 | **100** |  |
| Assebe, et al. | 2023 | 7 | 0 | 8 | 1 | 9 | 6 | 5 | 7 | 8 | 6 | 7 | 8 | 7 | 6 | 8 | 3 | **96** |  |
| Killedar, et al. | 2023 | 7 | 4 | 8 | 1 | 9 | 6 | 5 | 7 | 8 | 6 | 7 | 8 | 7 | 6 | 8 | 3 | **100** |  |
| Kwon, et al. | 2023 | 7 | 4 | 8 | 1 | 9 | 6 | 5 | 7 | 8 | 6 | 7 | 8 | 7 | 6 | 8 | 3 | **100** |  |
| Meunier, et al. | 2024 | 7 | 4 | 8 | 1 | 9 | 6 | 5 | 0 | 8 | 6 | 7 | 8 | 7 | 6 | 8 | 3 | **93** |  |
| Synnott, et al. | 2024 | 7 | 4 | 8 | 1 | 0 | 6 | 5 | 0 | 8 | 6 | 7 | 8 | 7 | 6 | 8 | 3 | **84** |  |
| Biundo, et al. | 2024 | 7 | 4 | 8 | 1 | 9 | 6 | 5 | 7 | 8 | 6 | 7 | 8 | 7 | 6 | 8 | 3 | **91** |  |
| Majda, et al. | 2025 | 7 | 4 | 8 | 1 | 9 | 6 | 5 | 7 | 8 | 6 | 7 | 8 | 7 | 6 | 8 | 3 | **100** |  |
| Walbaum, et al. | 2025 | 7 | 4 | 8 | 1 | 9 | 6 | 5 | 0 | 8 | 6 | 7 | 8 | 7 | 6 | 8 | 3 | **93** |  |
| Barry, et al. | 2025 | 7 | 4 | 8 | 1 | 9 | 6 | 5 | 7 | 8 | 6 | 7 | 8 | 7 | 6 | 8 | 3 | **100** |  |
| Tschampl, et al. | 2025 | 7 | 4 | 8 | 1 | 9 | 6 | 5 | 0 | 8 | 6 | 7 | 8 | 7 | 6 | 8 | 3 | **93** |  |
| Jansen, et al. | 2025 | 7 | 4 | 8 | 1 | 9 | 6 | 5 | 7 | 8 | 6 | 7 | 8 | 7 | 6 | 8 | 3 | **100** |  |
| Jansen, et al. | 2025 | 7 | 4 | 8 | 1 | 9 | 6 | 5 | 7 | 8 | 6 | 7 | 8 | 7 | 6 | 8 | 3 | **100** |  |
| Okafor, et al. | 2025 | 7 | 4 | 8 | 1 | 9 | 6 | 5 | 7 | 8 | 6 | 7 | 8 | 7 | 6 | 8 | 3 | **100** |  |
| Stanimirovic, et al. | 2025 | 7 | 4 | 8 | 1 | 9 | 0 | 5 | 7 | 8 | 6 | 7 | 8 | 7 | 6 | 8 | 3 | **94** |  |
| Kowal, et al. | 2025 | 7 | 4 | 8 | 1 | 9 | 6 | 5 | 7 | 8 | 6 | 7 | 8 | 7 | 6 | 8 | 3 | **100** |  |

**Table 7.** Study design and characteristics of the 28 included empirical studies that applied distributional cost-effectiveness analysis to evaluate the costs and health outcomes of at least two healthcare interventions, published from 2017 to 2025.

| **No.** | **Author** | **Publication Year** | **Study Regions** | **Research Perspective** | **Study Population** | **Disease Domain** | **Interventions and Controls** |
| --- | --- | --- | --- | --- | --- | --- | --- |
| 1 | Campos, et al. | 2017 | Africa | whole society | Adult women | Cancer | Expanding screening coverage; increasing screening frequency vs. no screening strategy |
| 2 | Dawkins, et al. | 2018 | Africa | health system | Children | Infectious Diseases | Standard vaccination program; pro-poor vaccination program vs. no vaccination |
| 3 | Lee, et al. | 2018 | Asia | whole society | Adult women | Cancer | Enhanced screening recommendations; universal screening recommendations vs. current strategy |
| 4 | Chang, et al. | 2018 | North America | health system | General population ; Patients | Cancer | New cancer therapies vs. SOC |
| 5 | Arnold, et al. | 2020 | Africa | health system | General population | Multiple Diseases | Malawi Basic Health Package vs. no EHP intervention |
| 6 | Collins, et al. | 2020 | Europe | health system | Adults | Cardiovascular Diseases | Current screening; enhanced universal screening; universal plus targeted screening vs. no cardiovascular disease screening |
| 7 | Love-Koh, et al. | 2020 | Europe | health system | Adults | Multiple Diseases | 21 smoking cessation interventions vs. no intervention |
| 8 | Love-Koh, et al. | 2021 | South America | health system | General population | Multiple Diseases | Brazil Family Health Program vs. pre-implementation |
| 9 | Quan, et al. | 2021 | North America | health system | Adults | Infectious Diseases | Allocation by baseline service ratio; allocation by new diagnosis ratio vs. current strategy |
| 10 | Olsen, et al. | 2021 | Africa | health system | Children | Infectious Diseases | CCM strategy vs. current strategy |
| 11 | Kowal, et al. | 2023 | North America | medical insurance payers | General population | Infectious Diseases | COVID-19 hospitalization treatment vs. SOC |
| 12 | Meunier, et al. | 2023 | Europe | health system | Patients | Cancer | Atezolizumab vs. docetaxel; alectinib vs. crizotinib |
| 13 | Goshua, et al. | 2023 | North America | health system | Patients | Hereditary hemoglobinopathies | Gene therapy vs. SOC |
| 14 | Assebe, et al. | 2023 | Africa | health system | General population | Infectious Diseases | 30 infectious disease interventions in EHSP vs. no intervention |
| 15 | Killedar, et al. | 2023 | Other | medical insurance payers | Children | Chronic metabolic diseases | POI-Sleep; POI-Combo; High Five for Kids vs. TAU |
| 16 | Kwon, et al. | 2023 | Europe | whole society | Elderly | Other | UK guideline recommended strategy vs. TAU |
| 17 | Meunier, et al. | 2024 | Europe | whole society | Patients | Cardiovascular Diseases | Faraxizumab vs. ranibizumab; aflibercept; bevacizumab |
| 18 | Stanimirovic, et al. | 2025 | North America | healthcare system | Patients | Other | Remote retinal screening vs. SOC |
| 19 | Synnott, et al. | 2024 | North America | whole society | General population | Neurodegenerative Diseases | Hypothetical Disease Modification Therapy vs. BSC |
| 20 | Kowal, et al. | 2025 | North America | medical insurance payers | Patients | Infectious Diseases | Tocilizumab vs. SOC |
| 21 | Biundo, et al. | 2024 | Europe | society; healthcare system; healthcare payers | General population | Infectious Diseases | Specific Vaccination Programs vs. No Vaccination Status |
| 22 | Majda, et al. | 2025 | North America | healthcare payers | General population | Cardiovascular Diseases | Alteplase vs. SOC |
| 23 | Walbaum, et al. | 2025 | South America | healthcare system | Adults; patients | Chronic Autoinflammatory Diseases | Etanercept; Peselilizumab; Infliximab; Adalimumab; Secukinumab; Golimumab vs. TAU |
| 24 | Barry, et al. | 2025 | North America | whole society | Adults | Cardiovascular Diseases | Medicare Expansion vs. No Medicare Expansion |
| 25 | Tschampl, et al. | 2025 | North America | whole society;  health system | Children | Other | Age Restriction Policy vs. No Age Restriction Policy |
| 26 | Jansen, et al. | 2025 | North America | healthcare system | Patients | Cancer | Darolulamide combined with ADT vs. ADT |
| 27 | Jansen, et al. | 2025 | North America | healthcare payers | Patients | Cancer | LB±TB vs. TB |
| 28 | Okafor, et al. | 2025 | Other | healthcare system | Adults | Infectious Diseases | Unit price XBB.1.5 Vaccine vs. No updated booster shot |

**Table 8.** Methodological characteristics and equity analysis outcomes of the 28 included empirical studies that applied distributional cost-effectiveness analysis to evaluate the costs and health outcomes of at least two healthcare interventions, published from 2017 to 2025.

| **No.** | **Author** | **Publication Year** | **Health effects** | **Subgroup Classification** | **Equity Index** | **Deterministic Sensitivity Analysis** | **Modeling Analysis** | **Basic-case cost-effectiveness results** | **Equity analysis results** |
| --- | --- | --- | --- | --- | --- | --- | --- | --- | --- |
| 1 | Campos, et al. | 2017 | Life expectancy | Accessibility and screening frequency | Not mentioned | Non-response rate | Monte Carlo Markov-chain micro-simulation models | Both expanding coverage and increasing frequency were cost-effective; icers: Expanding coverage to 50% at is150/yls; screening three times at 30% coverage at is540/yls; both below uganda's per capita gdp of is1,690 | Expanding coverage was win-win (quadrant i); increasing frequency was trade-off (quadrant ii) |
| 2 | Dawkins, et al. | 2018 | HALY | HWQ | Atkinson index | Opportunity cost distributions, inequality aversion parameters | Not mentioned | Standard programme was cost-effective compared to no vaccination; the pro-poor programme was not cost-effective compared to the standard programme (ICER: $69 per HALY, above the $50 threshold) | The pro-poor programme was trade-off (Quadrant IV) |
| 3 | Lee, et al. | 2018 | QALY | Geographic region | Atkinson index | not mentioned | Markov models | All strategies were cost-effective (ICERs: strong recommendation to target regions at KRW 7,361,145 per QALY; regular universal at KRW 8,584,172; strong universal at KRW 11,506,849; all below the Korean GDP threshold of KRW 29,901,550) | The targeted regional strategy was win-win (Quadrant I) |
| 4 | Chang, et al. | 2018 | QALY | race and ethnicity, SVI | Atkinson index | inequality aversion parameters, effectiveness and cost | Partitioned Survival Model | Vaccines were generally considered cost-effective based on prior evidence (ICERs not reported in this distributional analysis) | Vaccines were win-win (Quadrant I) |
| 5 | Arnold, et al. | 2020 | DALY | HWQ, IWI | Atkinson index | Inequality aversion parameters, disease incidence/prevalence, opportunity cost threshold | Not mentioned | The overall EHP was cost-effective (positive population net health benefit of 8.87 million DALYs averted) | The overall EHP was win-win (Quadrant I) |
| 6 | Collins, et al. | 2020 | QALY | IMD | Slope inequality index | Time range | Dynamic Stochastic Micro-Simulation Model | The universal plus targeted strategy was dominant compared with current screening and cost-effective compared with no screening (ICER: £1,500/QALY) | The universal plus targeted strategy reduced health inequality (ΔSII: -0.65 QALYs per 100,000) and was therefore win-win (Quadrant I) |
| 7 | Love-Koh, et al. | 2020 | QALY | IMD | Slope inequality index, relative inequality index, Atkinson index | Inequality aversion parameters, smoking cessation success rate | Markov models | All 21 interventions were either dominant (cost-saving) or cost-effective (ICER below £20,000/QALY) compared with no intervention | All interventions reduced the slope index of inequality (ΔSII positive) and increased equally distributed equivalent health, placing them in win-win (Quadrant I) |
| 8 | Love-Koh, et al. | 2021 | DALY | Geographic region, State-level economic development | Slope inequality index, relative inequality index, Atkinson index, Combe index | PSF cost | Markov models | The PSF was cost-effective with an average ICER of $2640 per DALY averted, below Brazil’s GDP per capita | The programme reduced health inequality between states (bivariate analysis showed pro-poor distribution) and improved social welfare, though the inequality reduction was small and uncertain; overall, it was win-win (Quadrant I) |
| 9 | Quan, et al. | 2021 | QALY | Race and ethnicity, HIV risk behaviors | Difference index, Theil index | Intervention cost adjustment | Dynamic HIV transmission model | The equity-focused implementation approach was dominant (cost-saving) or cost-effective (ICER below $100,000/QALY) compared with the proportional services approach | The equity approach reduced racial/ethnic health disparities across multiple inequality measures and increased population health, thus win-win (Quadrant I) |
| 10 | Olsen, et al. | 2021 | Life expectancy | Geographic region | Gini index | Not mentioned | Markov models | Scaling up community-based treatment to 90% coverage in all regions was cost-effective (ICERs: $26–199 per life year gained, below 50% of GDP per capita | The universal scale-up and health-maximizing strategies increased regional health inequality (Quadrant II), whereas the strategy prioritizing high-mortality regions reduced regional inequality but saved fewer lives (Quadrant IV). The universal scale-up was thus a trade-off (Quadrant II) |
| 11 | Kowal, et al. | 2023 | QALY | Race and ethnicity, SVI | Atkinson index | Opportunity cost threshold, inequality aversion parameters；opportunity cost distributions | Not mentioned | Funding inpatient COVID-19 treatments was cost-effective (ICER: $28,651/QALY, below $150,000/QALY threshold) | The intervention reduced health inequality (Atkinson index reduction of 0.234%), placing it in win-win (Quadrant I) |
| 12 | Meunier, et al. | 2023 | QALY | IMD | Atkinson index | Opportunity cost threshold, inequality aversion parameters, patient distribution | Not mentioned | Atezolizumab was not cost-effective at a £30,000/QALY threshold (ICER: £48,333/QALY, above threshold) but became cost-effective at a £50,000/QALY threshold; alectinib was cost-effective (ICER: £24,436/QALY, below £30,000/QALY threshold) | At a £30,000/QALY threshold, alectinib was win-win (Quadrant I, improving both health and equity); atezolizumab was trade-off (Quadrant IV, reducing health inequality at the cost of lower total health) at the same threshold, but became win-win at higher thresholds where it was cost-effective |
| 13 | Goshua, et al. | 2023 | QALY | Gender；disease severity | Atkinson index | Cost of gene therapy, age at which treatment begins, disease incidence/prevalence | Markov models | Gene therapy was not cost-effective compared with standard of care (ICER: $176,000 per QALY, above the $100,000/QALY threshold) | With inequality aversion parameters, gene therapy could be win-win (Quadrant I) if societal preferences for reducing disparities are sufficiently strong (threshold inequality aversion parameter of 0.90, within commonly used US ranges |
| 14 | Assebe, et al. | 2023 | HALY | HWQ | Slope inequality index, relative inequality index, Atkinson index, Combe index | Opportunity cost distributions, disease incidence/prevalence distributions, inequality aversion parameters | Not mentioned | The overall package was cost-effective (positive net health benefit of 2.29 million HALYs) | Overall, the package was win-win (Quadrant I); 23 interventions were win-win, 3 were trade-off (Quadrant II), and 4 were trade-off (Quadrant IV) |
| 15 | Killedar, et al. | 2023 | QALY | SEP/SEP | Slope inequality index | Assessment of individual heterogeneity | Socioeconomic status-specific micro-simulation models | POI-Sleep and H54K were cost-effective (positive net health benefit), while POI-Combo was not cost-effective (negative net health benefit) | POI-Sleep and H54K were win-win (Quadrant I, with 67% and 100% probability, respectively); POI-Combo was lose-lose (Quadrant III, with 91% probability) |
| 16 | Kwon, et al. | 2023 | QALY | SEP/SEP | Atkinson index | Not mentioned | Discrete Individual Simulation Model | Recommended care was cost-effective compared with usual care (societal ICER: £12,877–£15,149 per QALY gained, below £20,000/QALY threshold) | Recommended care was win-win (Quadrant I), with EDE incremental net health benefits exceeding the no-aversion benefit under both relative and absolute inequality aversion |
| 17 | Meunier, et al. | 2024 | QALY | IMD | Atkinson index | Opportunity cost threshold, inequality aversion parameters, perspective | Not mentioned | Faricimab was dominant compared with ranibizumab and aflibercept (cost-saving with QALY gains), and cost-effective compared with off-label bevacizumab (ICER implied by positive net health benefit below £20,000/QALY threshold) | Faricimab was win-win (Quadrant I), improving both health and reducing socioeconomic inequality across all comparators at the base-case opportunity cost threshold |
| 18 | Stanimirovic, et al. | 2025 | QALY | Income, educational attainment, and area-level deprivation, ethnicity, area-level ethnic diversity, gender, age | Atkinson index, Combe index, Gini index | Opportunity cost distributions | Decision Tree Model | Not applicable | Not applicable |
| 19 | Synnott, et al. | 2024 | QALY | Race and ethnicity, SVI | Atkinson index | Opportunity cost distributions, inequality aversion parameters, opportunity cost threshold | Markov models | The hypothetical disease-modifying treatment was cost-effective (ICER: $48,677/QALY, below $150,000/QALY threshold) | Treatment was win-win (Quadrant I), improving population health (28,197 QALYs gained) and reducing health inequality by 0.009% (Atkinson index reduction) |
| 20 | Kowal, et al. | 2025 | QALY | Race and ethnicity, SVI | Atkinson index | inequality aversion parameters, Opportunity cost threshold | Decision Tree-Markov models | Tocilizumab was cost-effective (ICER: $48,677/QALY, below $150,000/QALY threshold) | Treatment was win-win (Quadrant I), improving population health (53,252 QALYs gained) and reducing health inequality by 0.003% (Atkinson index reduction) |
| 21 | Biundo, et al. | 2024 | QALY | IMD | Combe index | hospitalization costs, length of hospital stay, inequality aversion parameters | Micro-simulation models | The rotavirus vaccination programme was cost-effective (baseline ICER: £23,337/QALY, below £20,000–£30,000 threshold). | With inequality aversion parameters, the intervention was win-win (Quadrant I), with 78% of prevented cases among the three most deprived groups and ICER reduced by 22–56% after equity weighting |
| 22 | Majda, et al. | 2025 | QALY | Race and ethnicity, SVI | Atkinson index | opportunity cost threshold, inequality aversion parameters, treatment rate scenario | Decision Tree-Markov models | Alteplase was dominant compared with standard of care (cost-saving with QALY gains; ICER not applicable as it was dominant) | Alteplase was win-win (Quadrant I), improving population health (45,606 QALYs gained) and reducing health inequality by 0.0001% annually |
| 23 | Walbaum, et al. | 2025 | QALY | SEP/SEP | Atkinson index | Inequality aversion parameters, disease incidence/prevalence distributions, opportunity cost threshold | Decision Tree-Markov models | The biologics were cost-effective (ICERs below 1–3 GDP per QALY thresholds, with Secukinumab having the most favourable ICER) | All biologics were win-win (Quadrant I), improving both health and equity, with Secukinumab showing the greatest positive impact on equity |
| 24 | Barry, et al. | 2025 | QALY | HWQ, Level of education, race and ethnicity | Atkinson index | Willingness to pay, inequality aversion parameters, household income, education, race/ethnicity | Monte Carlo Markov-chain micro-simulation models | Medicaid expansion was cost-effective (positive incremental net health benefit of 0.0031 QALYs per person at $150,000/QALY threshold, with 53% probability of being cost-effective) | Medicaid expansion was win-win (Quadrant I), improving health and reducing inequality across income and education groups, with 26–29% probability of being both cost-effective and equity-enhancing |
| 25 | Tschampl, et al. | 2025 | QALY | race and ethnicity, gender | not mentioned | Intervention efficacy rate, average duration of BED | Monte Carlo Markov-chain micro-simulation models | Age-restriction policy was dominant (cost-saving with QALY gains; ICER not applicable as it was cost-saving) | The policy was win-win (Quadrant I), improving health and reducing racial/ethnic health inequalities, with Latine residents experiencing the highest per capita health benefits (0.044 QALYs), followed by Black (0.042 QALYs) and White (0.038 QALYs) residents |
| 26 | Jansen, et al. | 2025 | QALY | race and ethnicity | Atkinson index | Inequality aversion parameters, opportunity cost threshold, race/ethnicity, survival differential adjustment | Individual Continuous-Time State Transition Model | Darolutamide + ADT was cost-effective compared with ADT alone (ICER: $146,754/QALY, below $150,000/QALY threshold) | Darolutamide was win-win (Quadrant I), reducing inequality in QALYs among patients by 66% and having a favourable impact on general population health inequality |
| 27 | Jansen, et al. | 2025 | QALY | race and ethnicity | Atkinson index, Combe index, Gini index | Opportunity cost threshold, inequality aversion parameters, performance adjustment for detection | Decision Tree-Partitioned Survival Model | Liquid biopsy-first strategy was cost-effective compared with tissue biopsy-only (incremental net health benefit of 91 QALYs per 100,000 general population at $150,000/QALY threshold) | Liquid biopsy-first was trade-off in the target patient population (Quadrant II), but was win-win in the general population (Quadrant I) |
| 28 | Okafor, et al. | 2025 | QALY | age；race and ethnicity | Not mentioned | Vaccine utilization rate, vaccine cost, symptom management cost, health utility, vaccine efficacy, transmission probability | Markov models | The updated vaccine was dominant for the 18-64 age group (ICER dominant), and cost-effective for the 65–74 and ≥75 age groups (ICERs: A$10,786/QALY and A$36,531/QALY, below A$50,000/QALY threshold) | The 18-64 group was likely win-win or neutral; the 65-74 and ≥75 groups were trade-off (Quadrant II |
